# Unveiling the Genetic and Physiological Synergies of Iron and Sulfur Homeostasis in Durum Wheat: From Root to Grain

**DOI:** 10.1111/ppl.70524

**Published:** 2025-09-18

**Authors:** Alessandro Bruschini, Eleonora Coppa, Giulia Quagliata, Miriam Marín‐Sanz, Andrea Ferrucci, Matteo Spada, Francesco Sestili, Francisco Barro, Gianpiero Vigani, Stefania Astolfi

**Affiliations:** ^1^ Department of Agricultural and Forestry Sciences (DAFNE) University of Tuscia Viterbo Italy; ^2^ Department of Plant Breeding Institute for Sustainable Agriculture (IAS), Spanish National Research Council (CSIC) Córdoba Spain; ^3^ Department of Life Sciences and Systems Biology University of Torino Italy

**Keywords:** Fe deficiency, genotyping‐by‐sequencing, ionome, phytosiderophores, sulfate assimilation

## Abstract

Fe deficiency is a major global challenge for agriculture. While high sulfur (S) supply can improve Fe nutrition in some grasses, the underlying mechanisms are poorly understood. This study investigated four genetically distinct durum wheat genotypes (Svevo, Karim, LcyE A^−^B^−^, and Svems16) to test the hypothesis that they employ different S‐mediated strategies to maintain Fe homeostasis under varying Fe availability. Fe deficiency inhibited plant growth and induced chlorosis with genotypic differences in severity. Notably, high S mitigated chlorosis in Karim and promoted root development in most genotypes, especially Svems16. Ionomic analysis showed that Fe deficiency primarily drove nutrient shifts in roots, but adding S restored shoot ionomic profiles. Total S analysis revealed genotype‐specific accumulation. Svevo showed consistently low S, possibly due to a sulfate transporter variant. Conversely, Karim exhibited elevated root S under combined stress, suggesting increased S channeling to phytosiderophore (PS) biosynthesis, supported by genotype‐dependent PS release. Genotyping‐by‐sequencing identified variants in methionine metabolism and PS‐related genes, offering molecular bases for the observed physiological differences. ATPS and OASTL activity patterns further confirmed the genotype‐specific role of root S metabolism in Fe deficiency response. Grain ionomics revealed that LcyE A^−^B^−^ enhanced Fe accumulation under combined high S and Fe deficiency, Svems16 under high S, while Karim, the most sensitive, had reduced grain Fe under deficiency. Our results highlight distinct, genotype‐specific strategies for maintaining Fe homeostasis and identify promising targets for breeding programs aimed at improving nutrient use efficiency and biofortification in durum wheat.

## 1 Introduction

The combined pressures of diminishing arable land and climate change urgently require a paradigm shift towards sustainable and efficient nutrient management in agriculture (UN 2017; Saleem et al. 2024). Iron (Fe) deficiency poses a significant challenge to global agriculture, severely compromising crop yield and nutritional quality, with direct consequences for food security and human health (Zuo and Zhang 2011). Iron deficiency in crops can significantly reduce its content in edible tissues, increasing the risk of Fe deficiency disorders in humans (MacDonald 2016). The World Health Organization (WHO) reported in 2019 that approximately 2 billion people suffer from anemia. This issue primarily affects groups with higher physiological Fe needs, such as children and women, especially in Africa and Southeast Asia, though it is also present in industrialized countries (WHO 2023). Increasing Fe content in the edible portions of cereal crops presents a major challenge, particularly given their status as a dietary staple for a significant portion of the global population. Their substantial nutritional contribution, including proteins, vitamins, and minerals, is indispensable for global food security and human health (McKevith 2004). Durum wheat, for instance, a key cereal crop in the Mediterranean region, provides approximately 20% of caloric and protein intake (Iqbal et al. 2022). Consequently, addressing Fe deficiency in this and other cereals offers a significant opportunity to combat anemia and improve grain nutritional value, particularly in vulnerable populations. This effort aligns with the overarching goal of achieving sustainable global food security, which necessitates the optimization of nutrient use efficiency (NutUE) through advanced agronomic techniques and the exploitation of synergistic nutrient interactions (Zhang et al. 2010). A growing body of research underscores the intricate interplay between sulfur (S) and Fe, revealing that deficiencies in either nutrient trigger compensatory physiological and/or metabolic adaptations aimed at maintaining balanced assimilation of the other (Astolfi et al. 2021). Specifically, S deficiency has been shown to limit Fe uptake and accumulation in grasses, such as barley, maize, and durum wheat (Astolfi et al. 2003; Bouranis et al. 2003; Astolfi, Cesco, et al. 2006; Zuchi et al. 2012), while Fe deficiency adaptation requires adjustments in S uptake and assimilation rates (Astolfi, Zuchi, et al. 2006; Ciaffi et al. 2013). Notably, supra‐optimal S supply has been observed to improve Fe use efficiency in wheat plants (Hawkesford et al. 2014) and to enhance shoot Fe accumulation by Fe‐deficient wheat plants (Zuchi et al. 2012). One possible explanation for these effects is that higher S availability may support the biosynthetic pathway of phytosiderophores (PS), as methionine serves as a common precursor for both PS and nicotianamine (Mori and Nishizawa 1987). Therefore, exploiting the Fe‐S interaction emerges as a promising strategy to enhance Fe uptake and accumulation in plants, contributing to the improvement of Fe concentration in food crops. Considering the aforementioned insights into the Fe/S interaction, this study aimed to investigate the variability in Fe accumulation responses to supra‐optimal S availability across four different durum wheat genotypes. Specifically, we examined two commercial cultivars, Svevo (Italian) and Karim (Tunisian), alongside two novel drought‐tolerant genotypes (LcyE A^−^B^−^ and Svems16) (Quagliata et al. 2023). Including drought‐tolerant genotypes in this research addresses the challenges posed by climate change and offers potential for practical applications in developing climate‐resilient and nutritionally enhanced crops. This study employed a two‐stage approach to comprehensively assess Fe accumulation in both vegetative tissues and grain. Initially, a controlled hydroponic experiment was conducted to evaluate plant S nutritional status and assimilation rate. Specifically, we determined: (i) total S and non‐protein thiol compound concentrations and (ii) ATP sulfurylase (ATPS) and O‐acetylserine(thiol)lyase (OASTL) enzyme activities. Also, the plant's capability to cope with Fe shortage was determined by measuring PS release in root exudates. The same genotypes were then grown in a greenhouse to assess grain Fe accumulation at full spike maturity. This dual approach facilitated the assessment of Fe dynamics across different growth stages and plant compartments. The findings are expected to facilitate the identification of durum wheat genotypes that, through optimized S nutrition, demonstrate enhanced Fe utilization efficiency and accumulation. Such genotypes would possess improved adaptability to growth‐limiting conditions, ultimately contributing to increased productivity and enhanced grain nutritional quality.

## 2 Results

### 2.1 Hydroponic Experiment

#### 2.1.1 Physiological and Morphological Responses of Durum Wheat Genotypes to Varying Fe and S Availability

**FIGURE 1:**
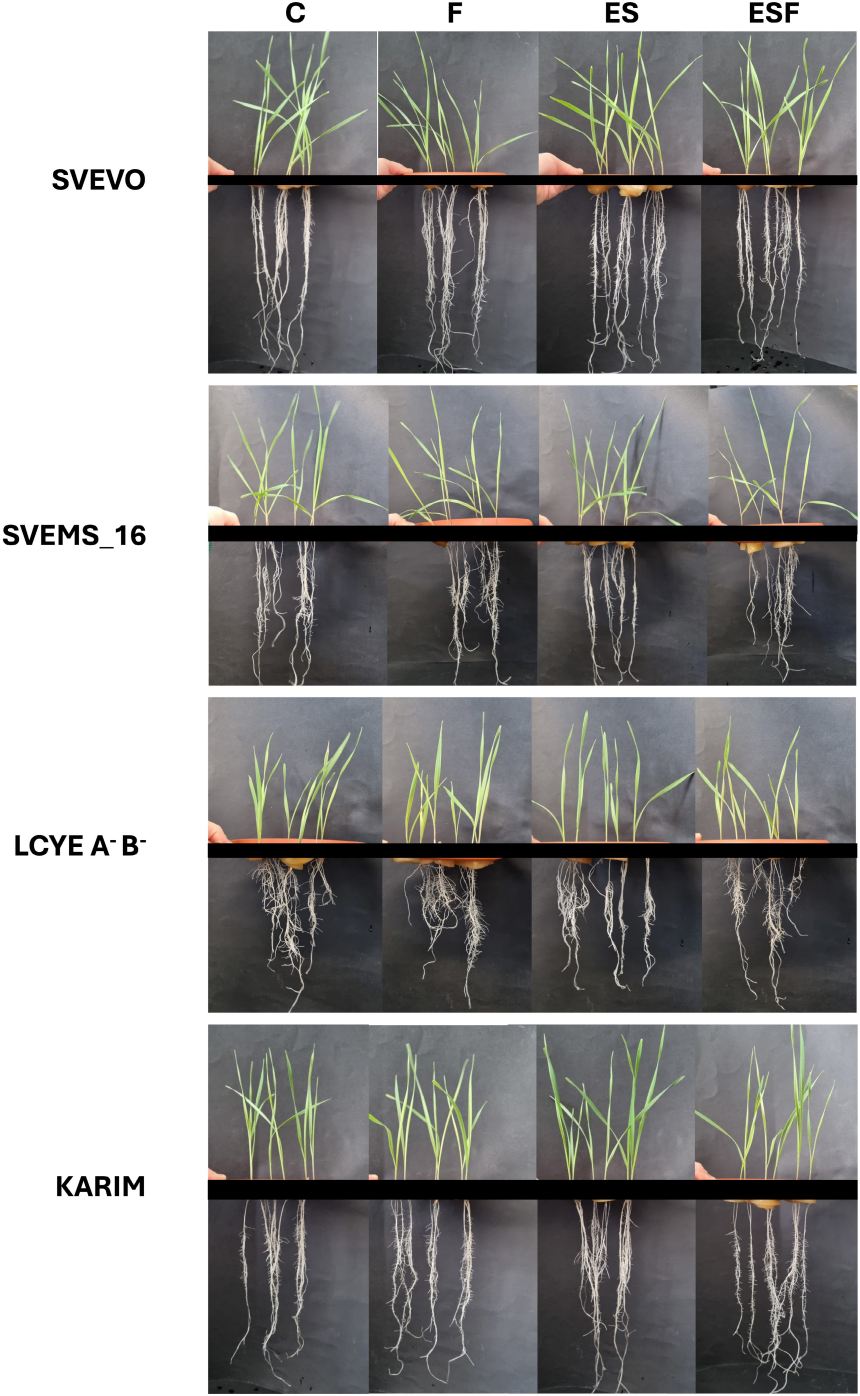
Comparison at harvest of four durum wheat ( *Triticum turgidum* subsp. *durum*) genotypes (Svevo, Svems16, LcyE A^−^B^−^, and Karim) grown hydroponically for 9 days under four different conditions: Control (C, 1.2 mM sulfate/80 μM FeIII‐EDTA), Fe deficiency (F, 1.2 mM sulfate/20 μM FeIII‐EDTA), supra‐optimal S (ES, 2.4 mM sulfate/80 μM FeIII‐EDTA), and combined supra‐optimal S and Fe deficiency (ESF, 2.4 mM sulfate/20 μM FeIII‐EDTA). The figure shows the phenotypic differences among genotypes in response to the treatments.

**FIGURE 2:**
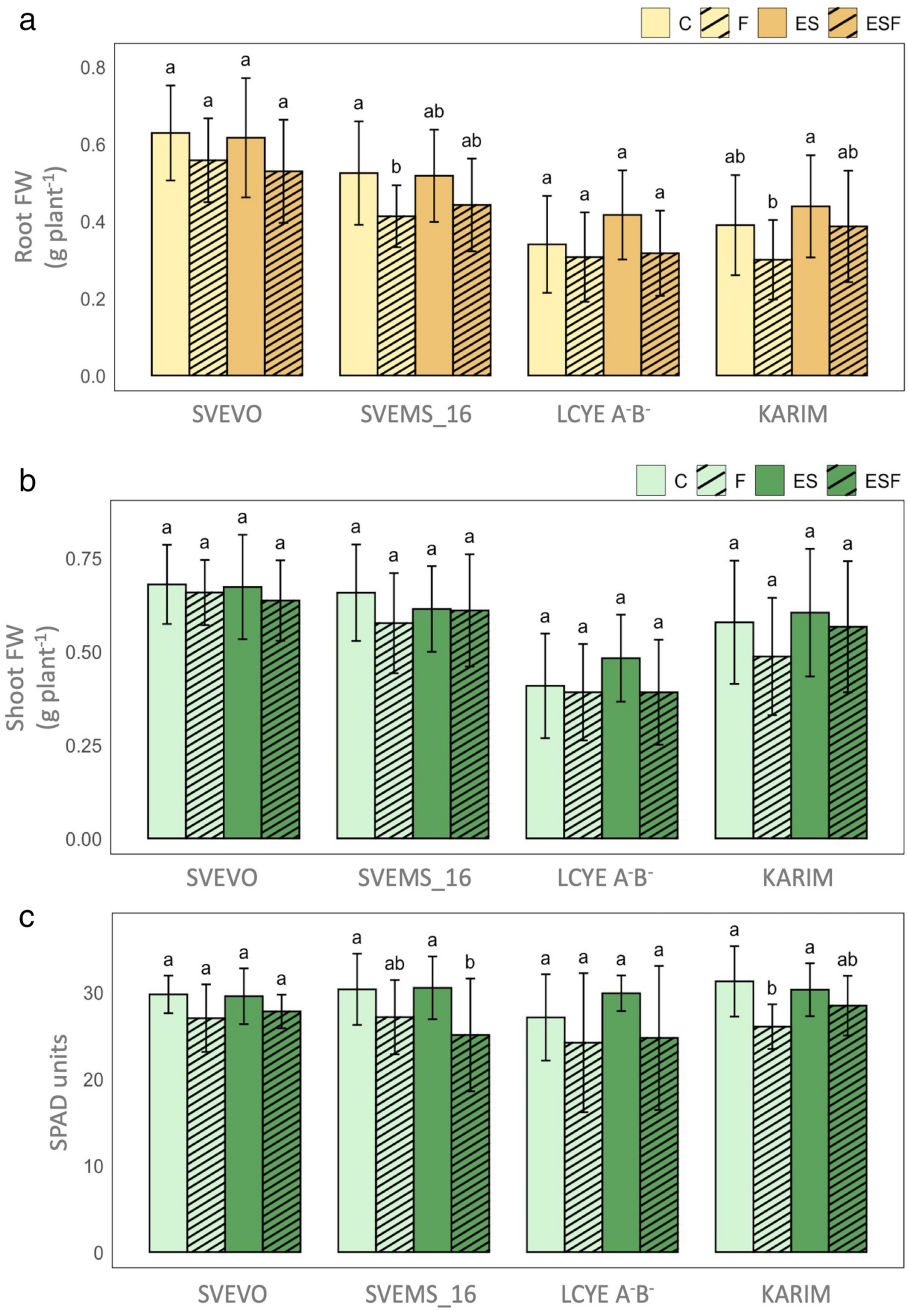
Fresh weight of roots (a) and shoots (b), and chlorophyll content (c) of plants of four durum wheat genotypes (Svevo, Svems16, LcyE A^−^B^−^, and Karim) grown hydroponically for 9 days under four different conditions: Control (C, 1.2 mM sulfate/80 μM FeIII‐EDTA), Fe deficiency (F, 1.2 mM sulfate/20 μM FeIII‐EDTA), supra‐optimal S (ES, 2.4 mM sulfate/80 μM FeIII‐EDTA), and combined supra‐optimal S and Fe deficiency (ESF, 2.4 mM sulfate/20 μM FeIII‐EDTA). Each reported value represents the mean ± standard deviation (SD) of measurements performed in triplicate and obtained from three independent biological replicates. Data were analyzed using two‐way ANOVA followed by one‐way ANOVA and Tukey's post hoc test (*p* < 0.05).

**FIGURE 3:**
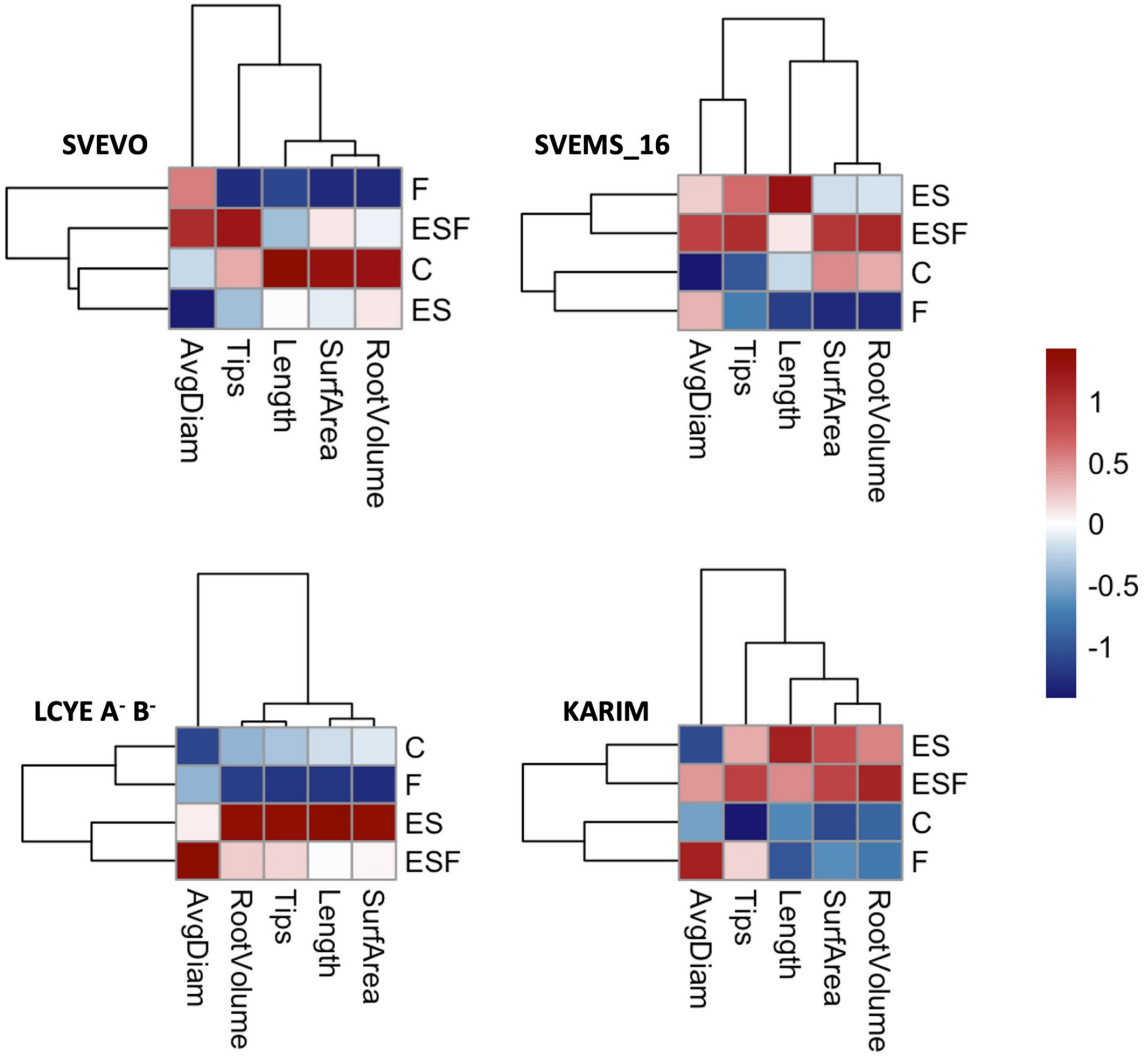
Heatmap representing hierarchical clustering of root traits dataset of four durum wheat genotypes (Svevo, Svems16, LcyE A^−^B^−^, and Karim) grown hydroponically as described in Figure 1. Statistics as in Figure 2. The color bar depicts the gradient of values of root traits.

The hydroponic experiment demonstrated that all treatments were well tolerated, with plants showing no significant damage symptoms during the experimental period (Figure 1). To evaluate genotypic variation in response to different growth conditions, we analyzed fresh root and shoot biomass, as well as chlorophyll content (Figure 2). Two‐way ANOVA revealed significant main effects of both growth conditions and genotype on root and shoot biomass (Table S1). However, the absence of significant interaction indicated consistent genotypic responses across conditions (Table S1). Svevo and Svems16 displayed the highest root and shoot biomass among the tested genotypes (Figure 2a,b). Fe deficiency (F) significantly reduced root biomass only in Svems16 (−22%) (Figure 2a). Notably, this reduction was mitigated when Fe deficiency was combined with S surplus (ESF), where Svems16 root biomass did not differ from the control (C) (Figure 2a). Shoot fresh weight remained unaffected by nutritional treatments in all four genotypes (Figure 2b). Two‐way ANOVA (Table S1) demonstrated that both treatment and genotype significantly influenced leaf chlorophyll content (Figure 2c). Fe deficiency (F) induced significant chlorosis only in the Karim genotype, resulting in an 18% chlorophyll reduction (Figure 2c). However, S supplementation associated with Fe deficiency (ESF) alleviated this effect, restoring chlorophyll levels in Karim to those of control plants (C) (Figure 2c). Conversely, Svems16 exhibited reduced chlorophyll content under ESF, with reductions of 16% and 15% compared to C and ES, respectively (Figure 2c). Root system architecture of each wheat genotype was analyzed, with root length, volume, diameter, surface area, and tip number quantified. Two‐way ANOVA (Table S1) revealed significant main effects of both growth condition and genotype on root architecture, along with significant interaction effects for all traits except average root diameter (Table S1). Heatmaps and hierarchical clustering were used to visualize root trait variations across growth conditions, facilitating pattern identification and data interpretation (Figure 3). Fe deficiency (F) induced a general reduction in most root architecture parameters in Svevo, Svems16, and LcyE A^−^B^−^, but had no significant effect on Karim (Figure 3). However, distinct genotypic responses were observed, indicating genotype‐specific root architectures differentially affected by growth conditions (Figure 3). Notably, root tip number significantly decreased only in Svevo and LcyE A^−^B^−^, remaining unchanged in Svems16 and Karim (Figure 3). Growth conditions also triggered specific changes with consistent genotypic response patterns. Specifically, ES and ESF conditions, as well as C and F ones, were clustered together in most genotypes. However, Svevo exhibited a unique response, with ESF clustering with C and ES (Figure 3a). Exposure to supra‐optimal S (ES) resulted in different root trait responses among genotypes. ES significantly increased root tip number in Svems16, LcyE A^−^B^−^, and Karim (Figures 3 and S1). This increase was also observed under ESF condition in Svevo (Figures 3a and S1). Additionally, ES enhanced root surface area in LcyE A^−^B^−^ and Karim, with this effect persisting in Karim under ESF (Figures 3c,d and S1). Similar trends were observed for root volume and total root length, with ES also promoting root length in Svems16 (Figures 3b and S1).

#### 2.1.2 Fe Deficiency and S Surplus Synergistically Modulate S Metabolism and Thiol Accumulation in Durum Wheat Genotypes

**FIGURE 4:**
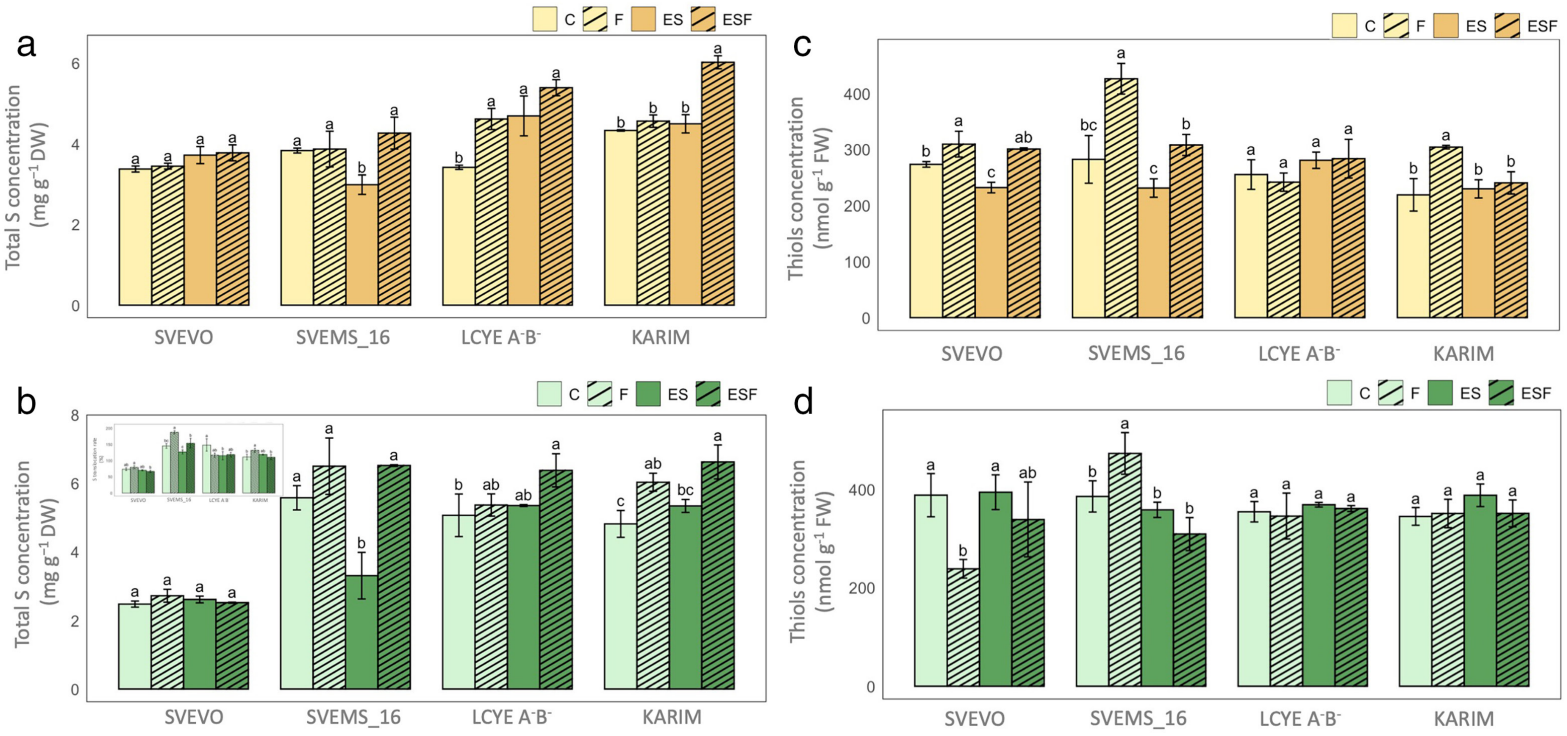
Total S concentration and thiols concentration in roots (a, c) and shoots (b, d) in plants of four durum wheat genotypes (Svevo, Svems16, LcyE A^−^B^−^, and Karim) grown hydroponically as described in Figure 1. Statistics as in Figure 2.

Two‐way ANOVA (Table S1) demonstrated significant main effects of both growth condition and genotype on total S concentration in root and shoot tissues (Figure 4a,b). A significant interaction effect indicated that genotypic responses to different growth conditions varied in terms of S accumulation (Table S1; Figure 4a,b). Notably, the combined Fe deficiency and S surplus (ESF condition) significantly increased S concentration in the roots of Karim and LcyE A^−^B^−^ genotypes. Karim exhibited a 38% increase, while LcyE A^−^B^−^ showed a 58% increase both compared to the control (C) (Figure 4a). In contrast, single Fe deficiency (F) and single S surplus (ES) did not significantly affect S accumulation in most genotypes, except for a substantial decrease in Svems16 roots under ES (Figure 4a). Similar trends were observed in shoot tissues (Figure 4b). The ESF condition significantly increased S concentration in Karim and LcyE A^−^B^−^ shoots, with Karim showing 38% and 24% increases compared to C and ES, respectively, and LcyE A^−^B^−^ exhibiting a 25% increase compared to C. Additionally, in Karim, single Fe deficiency (F) also led to a 25% increase in S accumulation compared to C (Figure 4b).

Thiols concentration in plant roots was significantly affected by both the growth condition and genotype, as revealed by the two‐way ANOVA analysis (Table S1). On the other hand, in shoot tissues, the growth condition factor had no significant effect (Table S1). A significant interaction between the two factors was detected at both the root and shoot levels (Table S1), suggesting that genotype influenced how thiol concentration responds to these varying growth conditions. Under Fe deficiency (F), all genotypes except LcyE A^−^B^−^ showed an increase in thiol content in the root tissues but to a different extent (Figure 4c). This increase was 13%, 51%, and 39% in Svevo, Svems16, and Karim, respectively, compared to control (C) (Figure 4c). The ES condition reduced thiol content only in Svevo (−15% vs. C), with no significant effects on other genotypes. Under ESF, thiol accumulation decreased in Svems16 and Karim compared to F (−28% and −21%, respectively), while it increased significantly in Svevo and Svems16 compared to ES (+30% and +33%, respectively) (Figure 4c). Thiol concentration in shoots (Figure 4d) remained largely unaffected by growth conditions, except for Svevo and Svems16, which respectively exhibited a significant decrease and increase under Fe deficiency (−40% and +23% vs. C) (Figure 4d).

**FIGURE 5:**
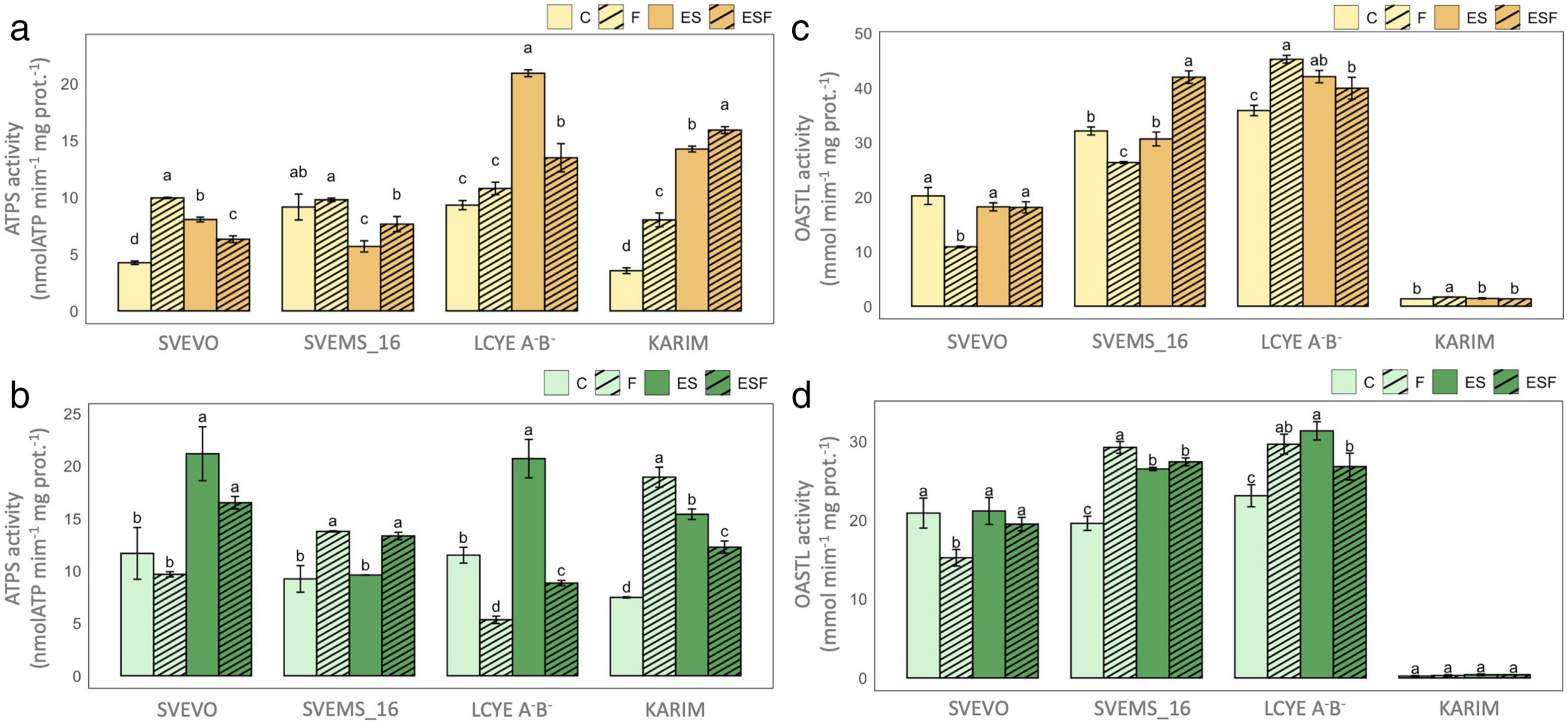
ATP sulfurylase (ATPS) and O‐acetylserine(thiol)lyase (OASTL) activity in roots (a, c) and shoots (b, d) in plants of four durum wheat genotypes (Svevo, Svems16, LcyE A^−^B^−^, and Karim) grown hydroponically as described in Figure 1. Statistics as in Figure 2.

We then analyzed the activities of key S metabolism enzymes, ATPS and OASTL (Figure 5). ATPS activity in both roots and shoots was significantly influenced by genotype, growth condition, and their interaction (Table S1), indicating genotype‐specific responses to different growth conditions. In roots (Figure 5a), Fe deficiency significantly increased ATPS activity in Svevo (+134% vs. C) and Karim (+126% vs. C). Excess S (ES) also increased ATPS activity in Svevo, LcyE A^−^B^−^, and Karim (+89%, +124%, and +303% vs. C, respectively), whereas an opposite behavior was observed in Svems16 (−38% vs. C). Under excess S combined with Fe deficiency (ESF), ATPS activity decreased in Svevo and LcyE A^−^B^−^ (−22% and −36% vs. ES, respectively), while it increased in Svems16 and Karim (+37% and +12% vs. ES, respectively). In shoots (Figure 5b), Fe deficiency had no significant effect on ATPS activity in Svevo but increased it in Svems16 and Karim (+49% and +154% vs. C, respectively) and decreased it in LcyE A^−^B^−^ (−53% vs. C). Excess S (ES) increased ATPS activity in Svevo, LcyE A^−^B^−^, and Karim (+81%, +80%, and +107% vs. C, respectively), but had no effect in Svems16. Excess S with Fe deficiency (ESF) decreased ATPS activity in Svevo, LcyE A^−^B^−^, and Karim (−20% to −57% vs. ES), but increased it in Svems16 (+39% vs. ES).

OASTL activity in both roots and shoots was significantly influenced by genotype, growth condition, and their interaction (Table S1), also suggesting genotype‐specific responses to different growth conditions. Notably, Karim exhibited significantly lower OASTL activity than all other genotypes in both root and shoot tissues (Figure 5c,d). In roots (Figure 5c), Fe deficiency (F) decreased OASTL activity in Svevo (−46%) and Svems16 (−18%) but increased it in LcyE A^−^B^−^ (+28%) and Karim (+21%) compared to control (C). Excess S (ES) only increased OASTL activity in LcyE A^−^B^−^ (+17%), while the combination of excess S with Fe deficiency (ESF) only increased it in Svems16 (+37% vs. ES). In shoots (Figure 5d), Fe deficiency significantly reduced OASTL activity in Svevo (−27% vs. C) but enhanced it in Svems16 (+49%) and LcyE A^−^B^−^ (+32%), with no effect observed in Karim. ES treatment increased OASTL activity in Svems16 (+36%) and LcyE A^−^B^−^ (+35%), but had no impact either on Svevo or Karim. Finally, there were no significant differences between ES and ESF conditions, except for a decrease in OASTL activity in LcyE A^−^B^−^ shoots (−13% vs. ES).

#### 2.1.3 Distinct Fe Accumulation and PS Exudation Responses Reveal Genotype‐Specific Adaptations to Nutrient Imbalances

**FIGURE 6:**
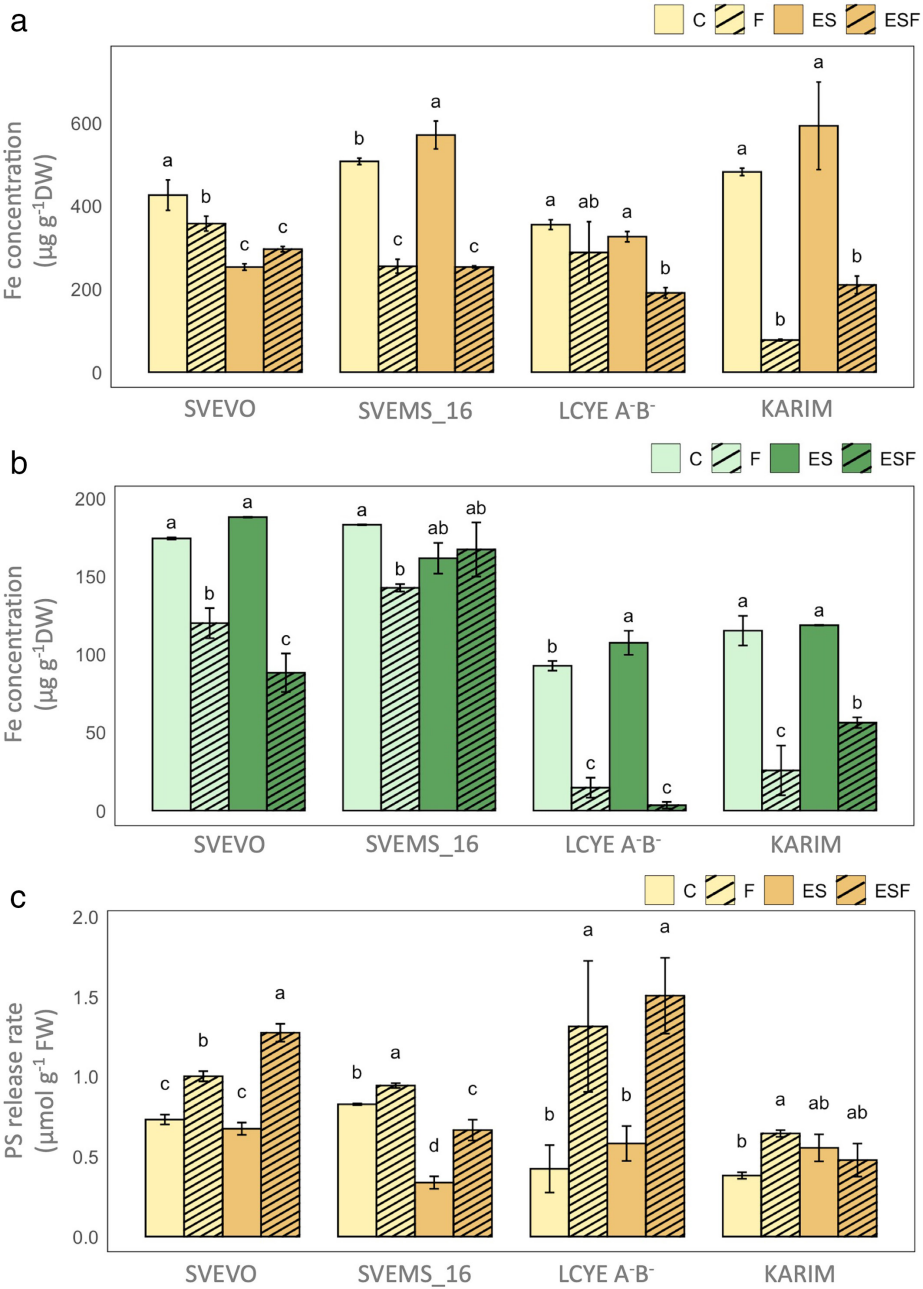
Fe concentration in roots (a) and shoots (b) and phytosiderophores (PS) release rate in plants (c) of four durum wheat genotypes (Svevo, Svems16, LcyE A^−^B^−^, and Karim) grown hydroponically as described in Figure 1. Statistics as in Figure 2.

Fe concentrations in roots and shoots were significantly influenced by genotype, growth condition, and their interaction (Table S1; Figure 6a,b), highlighting distinct genotype‐specific responses. In roots (Figure 6a), Fe deficiency (F) decreased Fe concentration in all genotypes except LcyE A^−^B^−^, with reductions of 16% in Svevo, 50% in Svems16, and 84% in Karim (the most sensitive). Excess S (ES) decreased Fe accumulation in Svevo (−41%) but increased it in Svems16 (+13). Under excess S with Fe deficiency (ESF), Fe concentration decreased similarly to the control (C) in Svems16 and Karim but further decreased in Svevo (−17% vs. Fe). In shoots (Figure 6b), Fe deficiency significantly reduced Fe concentration across all genotypes: 31% in Svevo, 22% in Svems16, 84% in LcyE A^−^B^−^, and 78% in Karim (again, the most sensitive). Excess sulfur (ES) increased Fe concentration only in LcyE A^−^B^−^ (+16% vs. C), while ESF promoted Fe accumulation, particularly in Karim (+119% vs. Fe). We then monitored PS release rate (Figure 6c). Two‐way ANOVA indicated significant effects of genotype, growth condition, and their interaction on PS release (Table S1). As expected, Fe deficiency increased PS release in all genotypes: 37% in Svevo, 69% in Karim, 210% in LcyE A^−^B^−^, and 14% in Svems16, compared to control (C). Excess S (ES) had no significant effect on PS release in most genotypes, except for a decrease in Svems16 (−59% vs. C). Under ESF, PS release further increased in Svevo compared to Fe deficiency but decreased in Svems16 (−29% vs. Fe) and remained unchanged in LcyE A^−^B^−^ and Karim.

#### 2.1.4 Genotypic Differences in Root and Shoot Mineral Nutrient Profile in Durum Wheat Under Differential Combination of Fe and S Availability

**FIGURE 7:**
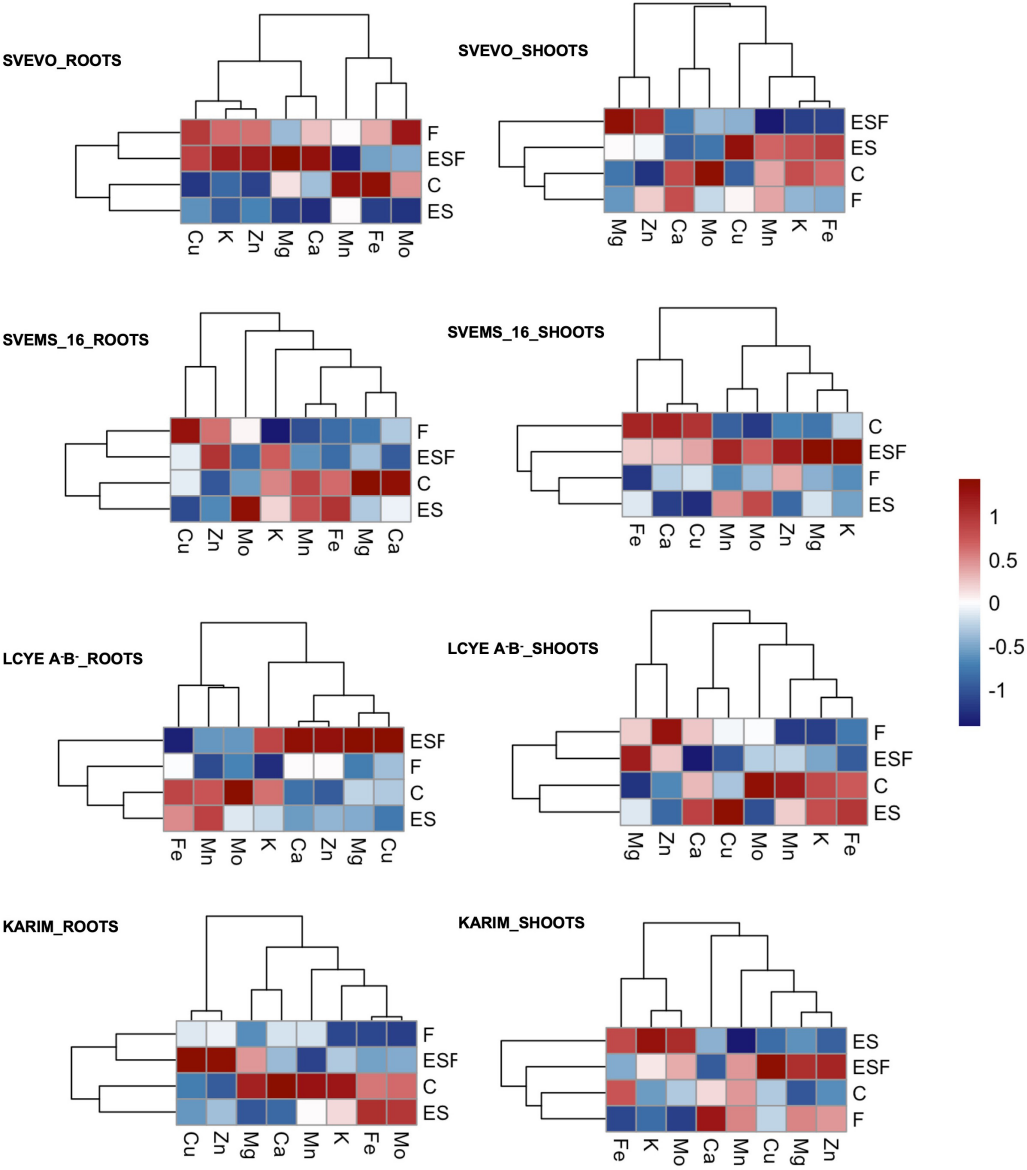
Heatmaps representing hierarchical clustering of root and shoot elemental composition of plants of four durum wheat genotypes (Svevo, Svems16, LcyE A^−^B^−^, and Karim) grown hydroponically as described in Figure 1. Color bar depicts relative ion concentrations across genotypes and treatments.

We analyzed the accumulation of potassium (K), magnesium (Mg), calcium (Ca), copper (Cu), zinc (Zn), manganese (Mn), and molybdenum (Mo) in both root and shoot tissues (Figures 7 and S2). Two‐way ANOVA revealed significant effects of genotype, growth condition, and their interaction on nutrient accumulation in most cases (Table S1). Exceptions were root K and shoot Cu, which were influenced only by genotype. Heatmaps and hierarchical clustering highlighted differences in root tissue nutrient accumulation across conditions (Figure 7). For most genotypes, F and ESF conditions showed similar patterns, distinct from control (C) and excess S (ES), indicating that Fe deficiency was the key driver of nutrient accumulation for these genotypes. However, LcyE A^−^B^−^ exhibited a unique pattern, with C, F, and ES clustering together and ESF alone. Moreover, genotype‐specific responses to the treatments were observed. In Svevo, F and ESF conditions led to increased K, Cu, Zn, and Ca accumulation, while ES treatment resulted in a general decline in nutrient concentrations, except for Mn. In Svems16, F and ESF conditions increased Cu and Zn levels but reduced the accumulation of Mn, Fe, Mg, and Ca. In Karim, both F and ESF conditions caused a decreased Mo, Fe, K, Mn, and Ca accumulation. In LcyE A^−^B^−^, while the ESF condition enhanced the accumulation of Cu, Mg, Zn, Ca, and K, F alone resulted in a reduction of most nutrient levels. While root tissues showed a clustering pattern more directly linked to the applied nutritional treatments, the clustering of growth conditions in shoot tissues was markedly more complex (Figure 7). In Svevo, ESF condition clustered alone and induced decreased accumulation of most nutrients, except for Zn and Mg, which increased. In Svems16, the control (C) condition clustered alone, and Fe deficiency (F), excess S (ES), and excess S with Fe deficiency (ESF) similarly altered accumulation patterns. Specifically, ESF increased the accumulation of all considered nutrients, ES increased only Mn and Mg, and F increased only Zn accumulation. In LcyE A^−^B^−^, Fe deficiency (F) and excess S with Fe deficiency (ESF) showed similar patterns, distinct from control (C) and excess S (ES), indicating that Fe deficiency was the key driver of nutrient accumulation for this genotype. F and ESF conditions decreased the accumulation of most nutrients but increased that of Mg and Zn. Finally, in Karim, ES condition clustered alone, showing decreased Mn and Ca and increased K accumulation. Interestingly, both conditions characterized by Fe deficiency (F and ESF) clustered together with the control (C) condition.

### 2.2 Greenhouse Experiment

#### 2.2.1 Monitoring Leaf Chlorophyll Content and Yield Traits Highlights Genotype‐Specific S‐Mediated Adaptations to Fe Deficiency

Throughout the greenhouse experiment, we recorded leaf chlorophyll content and, at harvest, three yield‐related traits, such as 1000‐grain weight, spike weight, and spike number (Table 1). Chlorophyll content was monitored at 22, 36, 49, and 63 days after transplanting (DAT) (Table 1). Two‐way ANOVA revealed that both growth conditions and genotype significantly influenced chlorophyll content up to 49 DAT, while at 63 DAT, only genotype had a significant effect. The interaction between these factors remained significant throughout (Table S2). Svevo plants subjected to Fe deficiency (F) displayed initial chlorosis, characterized by a 12% reduction in chlorophyll content relative to control plants. Notably, plants grown under ESF condition recovered rapidly, showing chlorophyll levels comparable to the control (Table 1). The chlorosis symptom gradually diminished, and by the penultimate measurement, Fe‐deficient plants exhibited a 10% increase in chlorophyll compared to the control. Excess S (ES) treatment induced a transient 10% chlorophyll reduction at the second time point, followed by a recovery to control levels. At the final measurement, chlorophyll content was statistically similar across all conditions (Table 1). In Svems16, chlorosis appeared in condition F at 36 DAT but recovered to control levels by 63 DAT. Notably, the ESF condition resulted in the highest chlorophyll content, exceeding the control by 10%. For LcyE A^−^B^−^, chlorosis was observed later at 49 DAT, also linked to high S availability (ES) (Table 1). At the end of the experiment (63 DAT), plants in condition F showed a 12% reduction in chlorophyll compared to the control, whereas plants in the ES condition returned to control chlorophyll levels (Table 1). The response in Karim was notably different. At 22 DAT, chlorophyll content was lower across all tested conditions (F −18%, ES −27%, ESF −27%). However, at 36 DAT, plants in condition F exhibited the highest chlorophyll content, with a 15% increase compared to the control (Table 1). By 49 DAT, plants in both ES and ESF conditions also exceeded control levels by 15%. Nevertheless, by the final measurement at 63 DAT, the chlorophyll content across all conditions was statistically similar to that of the control plants (Table 1).

**TABLE 1:** One thousand‐grain weight, spike weight, spike number, and chlorophyll content 22, 36, 49 and 63 days after transplanting (DAT), measured in four different tetraploid wheat genotypes from the greenhouse experiment under different nutrient availability conditions: C, control; F, Fe‐deficiency; ES, supra‐optimal S; ESF, supra‐optimal S/Fe‐deficiency.

Genotype	Growth condition	One thousand seeds weight (g seeds^−1^ DW/n° seeds)	Spikes weight (g^−1^ DW)	Number of spikes	Chlorophyll content 9 February (SPAD unit)	Chlorophyll content 23 February (SPAD unit)	Chlorophyll content 8 March (SPAD unit)	Chlorophyll content 22 March (SPAD unit)
Svevo	C	43.59 ± 3.45 a	1.16 ± 0.28 ab	1.13 ± 0.11 ns	43.34 ± 2.18 a	40.42 ± 1.87 a	42.9 ± 2.21 b	42.85 ± 2.77 ns
	F	45.20 ± 2.23 a	1.19 ± 0.29 ab	1 ± 0.1 ns	37.08 ± 5.12 b	40.93 ± 2.39 a	46.17 ± 2 a	45.91 ± 3.98 ns
	ES	43.69 ± 4.38 a	0.81 ± 0.45 b	1.22 ± 0.12 ns	42.09 ± 1.39 a	36.78 ± 2.14 b	41.14 ± 1.13 b	44.20 ± 4.53 ns
	ESF	37.23 ± 2.68 b	1.37 ± 0.19 a	1 ± 0.1 ns	39.09 ± 2.14 ab	42.32 ± 2.20 a	45.97 ± 1.8 a	46.24 ± 3.45 ns
Svems 16	C	41.66 ± 2.70 ns	0.78 ± 0.43 b	1.67 ± 0.17 ns	32.45 ± 2.19 b	37.43 ± 1.69 a	38.63 ± 2.61 ns	38.97 ± 1.99 b
	F	36.54 ± 2.68 ns	1.07 ± 0.34 ab	1.57 ± 0.16 ns	40.35 ± 4.27 a	32.58 ± 1.35 b	38 ± 2.5 ns	40.03 ± 2.02 ab
	ES	40.77 ± 4.29 ns	0.77 ± 0.54 b	1.57 ± 0.16 ns	31.37 ± 0.45 b	31.75 ± 1.97 b	37.28 ± 1.49 ns	40.82 ± 2.16 ab
	ESF	39.20 ± 2.75 ns	1.09 ± 0.51 a	1.57 ± 0.16 ns	38.58 ± 2.10 a	36.45 ± 2.15 a	41.4 ± 1.81 ns	42.75 ± 1.93 a
LcyE A^−^B^−^	C	39.07 ± 2.84 ns	1.12 ± 02 a	1.5 ± 0.15 ns	32.07 ± 2.32 b	36.25 ± 0.65 ns	45.38 ± 2.56 a	47.68 ± 3.48 ab
	F	45.09 ± 3.47 ns	0.77 ± 0.21 b	1.17 ± 0.12 ns	36.83 ± 1.67 a	38.95 ± 1.34 ns	40.68 ± 2.43 b	41.83 ± 5.25 b
	ES	37.33 ± 3.63 ns	0.91 ± 0.20 b	1.50 ± 0.15 ns	28.30 ± 1.70 b	37.37 ± 2.61 ns	39.93 ± 0.55 b	49.8 ± 0.4 ab
	ESF	46.57 ± 2.96 ns	1.02 ± 0.49 a	1.40 ± 0.14 ns	28.47 ± 1.89 b	35.30 ± 2.99 ns	42.55 ± 5.36 a	49.98 ± 3.65 a
Karim	C	44.75 ± 1.96 ns	1.62 ± 0.33 ns	1.25 ± 0.13 ns	41 ± 2.2 a	38.17 ± 3.39 b	40.36 ± 1.42 b	45.73 ± 2.62 ab
	F	46.20 ± 2.68 ns	1.69 ± 0.15 ns	1 ± 0.1 ns	34.8 ± 2 b	44.93 ± 2 a	48.39 ± 2.65 a	48.38 ± 4.54 a
	ES	46.57 ± 3.60 ns	1.52 ± 0.28 ns	1 ± 0.1 ns	30.9 ± 2.1 c	39.94 ± 1.63 b	46.74 ± 1.57 a	44.83 ± 3.56 ab
	ESF	47.89 ± 3.10 ns	1.38 ± 0.31 ns	1.13 ± 0.11 ns	31.7 ± 2.4 c	38.23 ± 2.28 b	46.08 ± 0.9 a	42.66 ± 3.43 b

*Note:* Each reported value represents the mean ± standard deviation (SD) of measurements performed in triplicate and obtained from three independent biological replicates. Data were analysed using two‐way ANOVA followed by one‐way ANOVA and Tukey's post hoc test (*p* < 0.05).

Statistical analysis using two‐way ANOVA (Table S2) demonstrated that 1000‐seed weight and number of spikes per plant were significantly influenced only by genotype, with no significant effect of growth conditions. However, spike weight exhibited significant effects from both genotype and growth conditions. The interaction between genotype and growth conditions was statistically significant for 1000‐seed weight and number of spikes, but not for spike weight. In Svevo plants, 1000‐seed weight significantly decreased only under the ESF condition (Table 1). Conversely, spike weight significantly increased under the ESF condition, but only when compared to ES treatment (+69%). No significant changes were observed in the number of spikes (Table 1). In Svems16, the only significant change was a 37% increase in spike weight under F condition compared to control (C), while 1000‐seed weight and number of spikes were unaffected by the treatments (Table 1). LcyE A^−^B^−^ showed an opposite trend to Svems16: spike weight significantly decreased under Fe deficiency (F) (−31% vs. C). However, the ESF condition led to significantly increased spike weight (+12% vs. ES), though it did not exceed control values. Thousand‐seed weight and the number of spikes remained unchanged in this genotype (Table 1). Finally, in Karim plants, none of the three traits showed significant variations under the tested conditions (Table 1).

**FIGURE 8:**
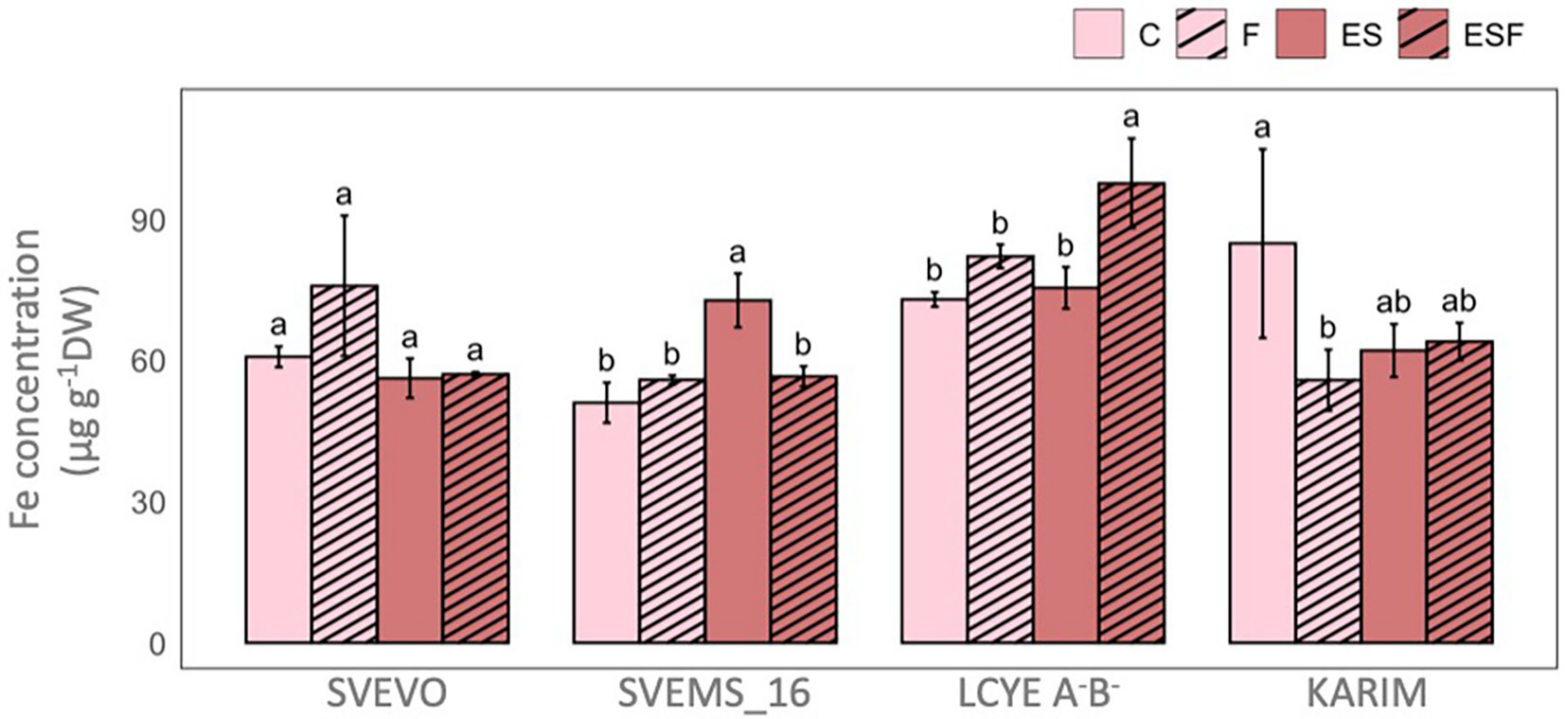
Fe concentration in the grain of four durum wheat genotypes (Svevo, Svems16, LcyE A^−^B^−^, and Karim) grown in the greenhouse. Growth conditions and statistics as in Figure 2.

Grain Fe accumulation was significantly influenced by genotype, with distinct responses to growth conditions, as shown by two‐way ANOVA (Table S1). A significant genotype‐by‐condition interaction was also noted. According to what was observed for the chlorophyll content, Svems16 demonstrated increased Fe accumulation under ES condition (+42% vs. C). LcyE A^−^B^−^ accumulated Fe only under ESF condition, showing increases of 33% versus control (C) and 30% versus ES. Conversely, Karim grain Fe levels significantly decreased by 34% only under F condition (Figure 8).

#### 2.2.2 Genotype‐Specific Patterns of Mineral Nutrient Content in Durum Wheat Grain Under Fe Deficiency and S Surplus

**FIGURE 9:**
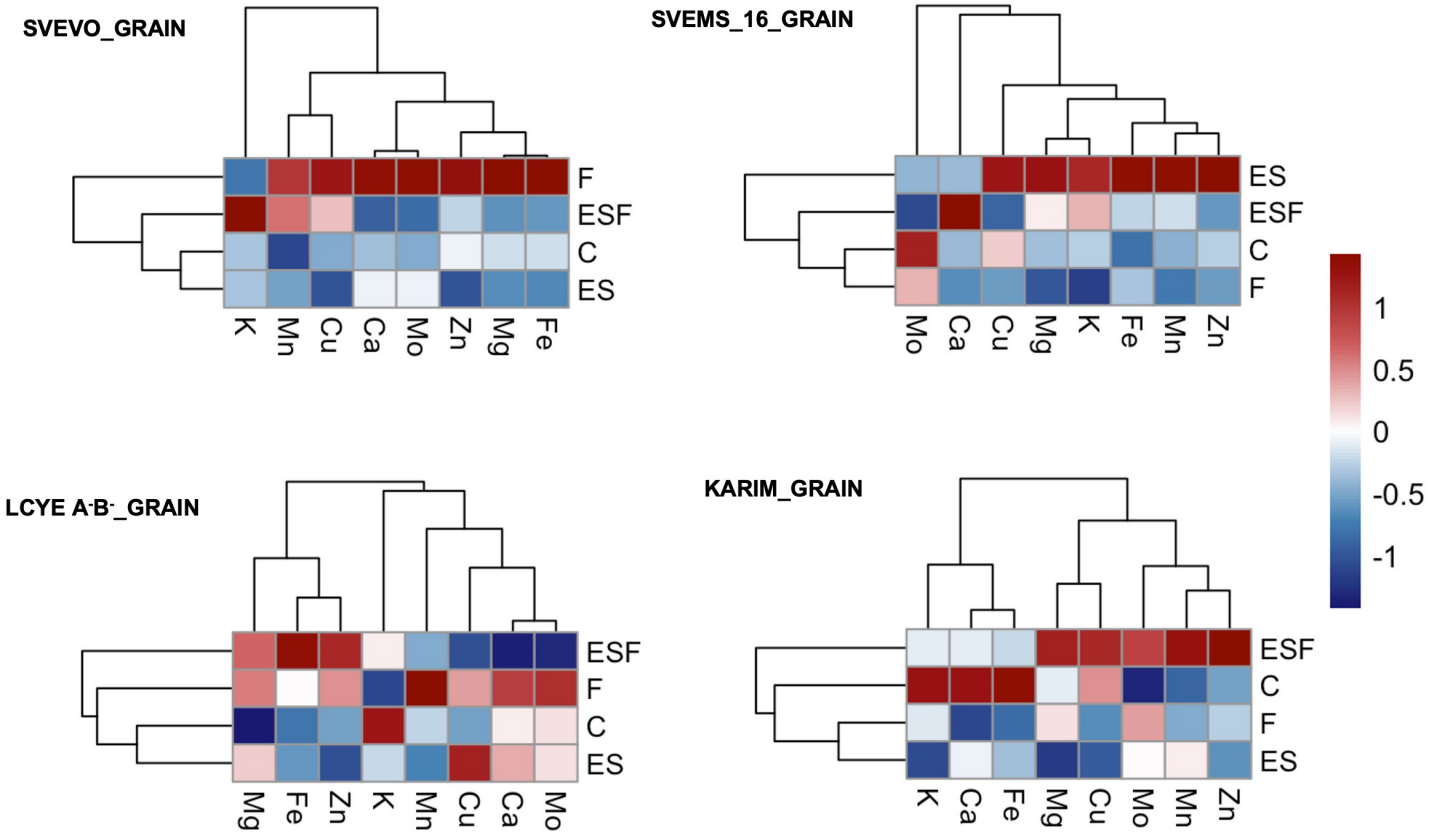
Heatmaps representing hierarchical clustering of grain elemental composition of four durum wheat genotypes (Svevo, Svems16, LcyE A^−^B^−^, and Karim) grown in a greenhouse. Growth conditions are described in Figure 1. Color bar depicts relative ion concentrations across genotypes and treatments.

Grain accumulation of K, Mg, Ca, Cu, Zn, Mn, and Mo was monitored at harvest (Figure S2). Two‐way ANOVA (Table S1) indicated significant effects of both growth condition and genotype, as well as their interaction, on the accumulation of these nutrients (Table S2). Heatmaps and hierarchical clustering were used to visualize accumulation patterns across growth conditions (Figure 9). Genotype‐specific accumulation patterns were observed. In Svevo, single Fe deficiency (F) drove the accumulation of most nutrients, with control (C), excess S (ES), and excess S with Fe deficiency (ESF) clustering separately (Figure 9a). In Svems16, excess S (ES) promoted nutrient accumulation, while other conditions clustered together (Figure 9b). Notably, when Fe deficiency was combined with excess S (ESF condition), the beneficial effect of excess S was nullified (Figure 9b). Accumulation patterns in LcyE A^−^B^−^ and Karim were largely comparable, with ESF condition showing a distinct clustering, while C, F, and ES clustered together (Figure 9c,d). Specifically, in Svevo, Fe deficiency (F) resulted in increased Mg (19%), Mn (38%), Cu (25%), and Mo (44%) compared to control (C). ESF condition increased the accumulation of K (15% vs. C and ES), Mn (31% vs. C, 18% vs. ES), and Cu (20% vs. ES). In Svems16, ES condition increased Mg (19%), Mn (51%), and Zn (58%) levels compared to C. In LcyE A^−^B^−^, Fe deficiency (F) increased the accumulation of Mg (22%), Ca (16%), Mn (20%), Zn (18%), and Mo (27%) compared to C. Both ES and ESF conditions raised the Mg level by approximately 20%. ESF condition increased Zn level (30% vs. C, 42% vs. ES) but decreased Ca (28%, 31% vs. C and ES) and Mo (43% vs. C and ES). In Karim, all nutritional treatments increased Mo accumulation (28%, 37%, 62% for F, ES, and EFS condition vs. C, respectively) and decreased K accumulation (8%, 14%, 8% for F, ES, and EFS condition vs. C, respectively). Fe deficiency (F) also decreased Ca accumulation (27% vs. C), while ESF condition increased that of Mn (22% vs. C).

### 2.3 Genome‐Wide Variants of the Four Genotypes

#### 2.3.1 Genome‐Wide Variants Affecting Functional Profiles of the Four Genotypes

**FIGURE 10:**
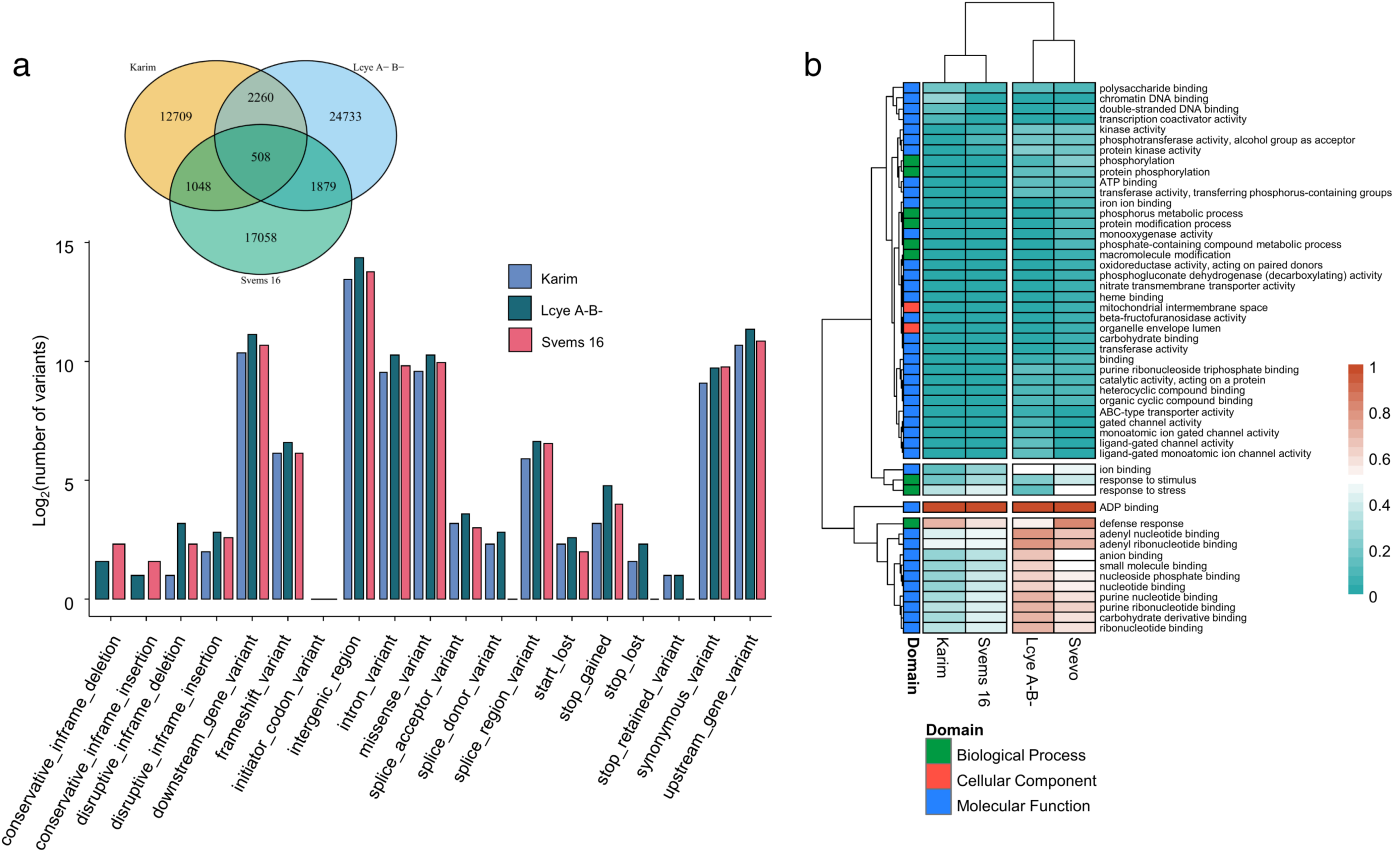
(a) Venn diagram showing the number of shared variants among the three durum wheat genotypes, Karim, LcyE A^−^B^−^ and Svems 16 compared to the reference genome Svevo, and the percentage of SNPs, insertions, and deletions identified in each genotype. The bar plot displays the logarithm of the number of variants (SNPs + InDels) per predicted effect in the same genotype. (b) Gene Ontology (GO) enrichment analysis of the genes containing variants in Karim, Svems 16, LcyE A^−^B^−^ and Svevo.

The genome‐wide variant profiles obtained from the GBS analysis revealed clear differences among the four genotypes. We then further analyzed variants in LcyE A^−^B^−^, Svems16, and Karim that were not present in the Svevo reference genotype. These genotypes exhibited distinct variant patterns when categorized by their predicted effects (Figure 10a). Notably, LcyE A^−^B^−^ harbored a higher number of variants than both Karim and Svems16, sharing more variants with Karim than with Svems16, as shown in the Venn diagram (Figure 10a). Most of the variants across all three genotypes were classified as intergenic, particularly in LcyE A^−^B^−^, reflecting their location outside of annotated genes. A significant proportion of variants was also found within 5000 bp upstream and downstream coding regions, referred to as upstream gene variants and downstream gene variants, respectively, suggesting potential regulatory effects on gene expression (Figure 10a). Furthermore, an important number of variants resulted in missense and synonymous mutations within coding regions, indicating possible alterations in protein function. InDels leading to frameshifts were also detected in all three genotypes. Importantly, high‐impact variants, such as stop‐lost mutations, were commonly observed in Karim and LcyE A^−^B^−^, but less common in Svems16 (Figure 10a). To explore the functional implications of these coding‐region variants, a GO enrichment analysis was conducted. The enriched GO terms, visualized by −Log_10_(Padj) values, revealed two distinct clusters: one grouping Svevo and LcyE A^−^B^−^, and another comprising Svems16 and Karim (Figure 10b). Within the first cluster, a subcluster exhibited enriched GO terms such as anion binding and carbohydrate derivative binding, distinguishing it from the second cluster (Figure 10b).

#### 2.3.2 Fe/S‐Related Gene Variants in the Four Genotypes

**FIGURE 11:**
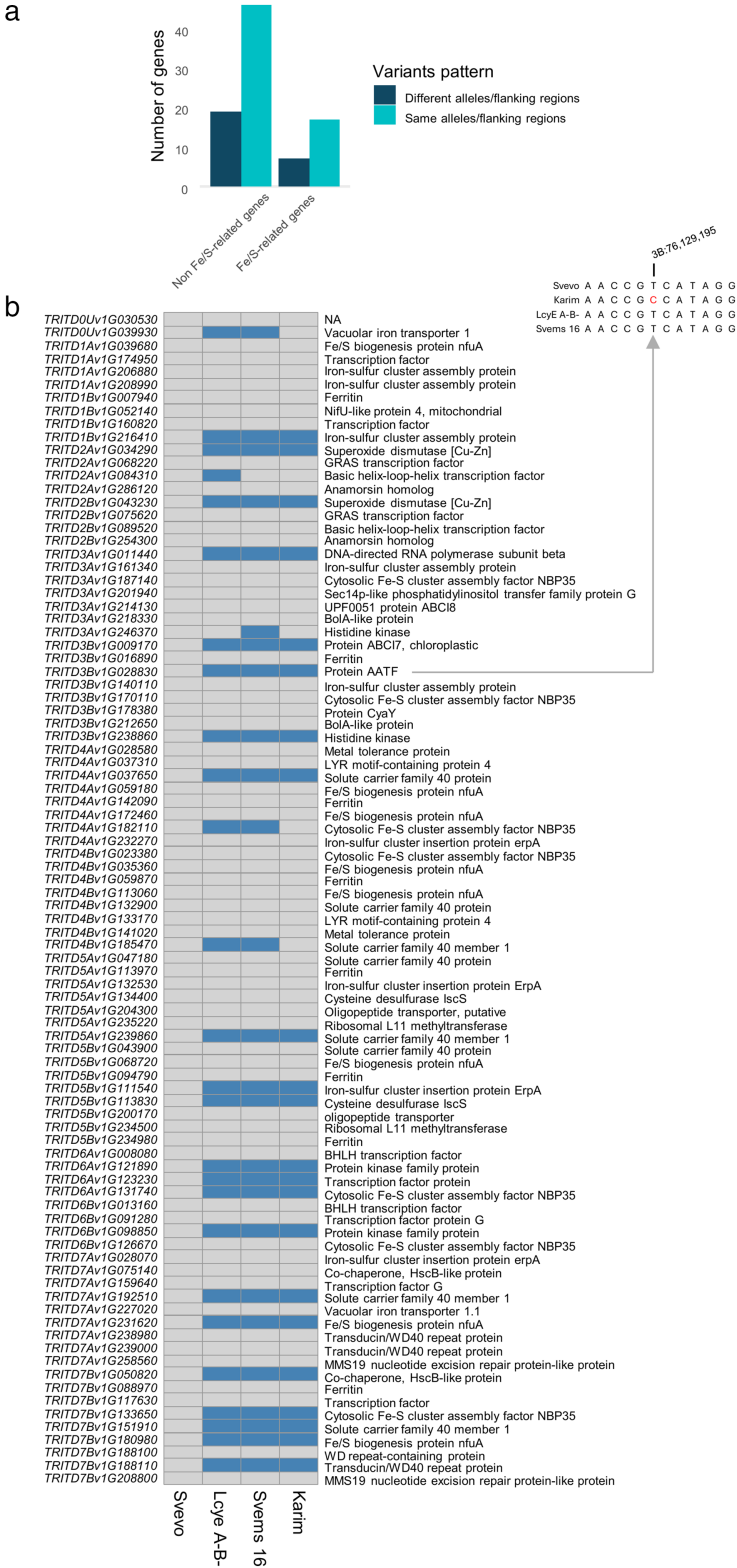
(a) Number of genes containing genetic variants based on the localization of polymorphisms (coding region, flanking region, or both) and allelic pattern (conserved vs. divergent alleles) among four genotypes: Svevo, LcyE A^−^B^−^, Svems16, and Karim. (b) Heatmap showing genotype‐specific sequence variations in genes associated with iron‐related functions. Blue cells indicate missense variants, while gray cells indicate downstream variants.

To specifically investigate variants in Fe‐related genes, we filtered the gene set using Biological Process GO terms related to Fe, including Fe‐S cluster assembly (GO:0016226), Fe ion transmembrane transport (GO:0034755), or Fe ion homeostasis (GO:0055072) (Table S3). Many of the filtered genes were involved in Fe/S cluster formation, encoding proteins associated with Fe‐S biogenesis (Figure 11a,b). As shown in Figure 11, approximately 70.8% of these genes shared the same alleles across all four genotypes, with no alterations in their flanking regions. However, several genes displayed genotype‐specific sequence variations. For instance, the gene TRITD7Bv1G151910, encoding Solute Carrier Family 40 member 1 (involved in Fe ion transmembrane transport), exhibited a missense variant in LcyE A^−^B^−^, Svems 16, and Karim, relative to Svevo. Meanwhile, another copy of the same gene, TRITD4Bv1G185470, showed a downstream variant in LcyE A^−^B^−^ and Svems 16, potentially affecting gene regulation (Figure 11b).

**FIGURE 12:**
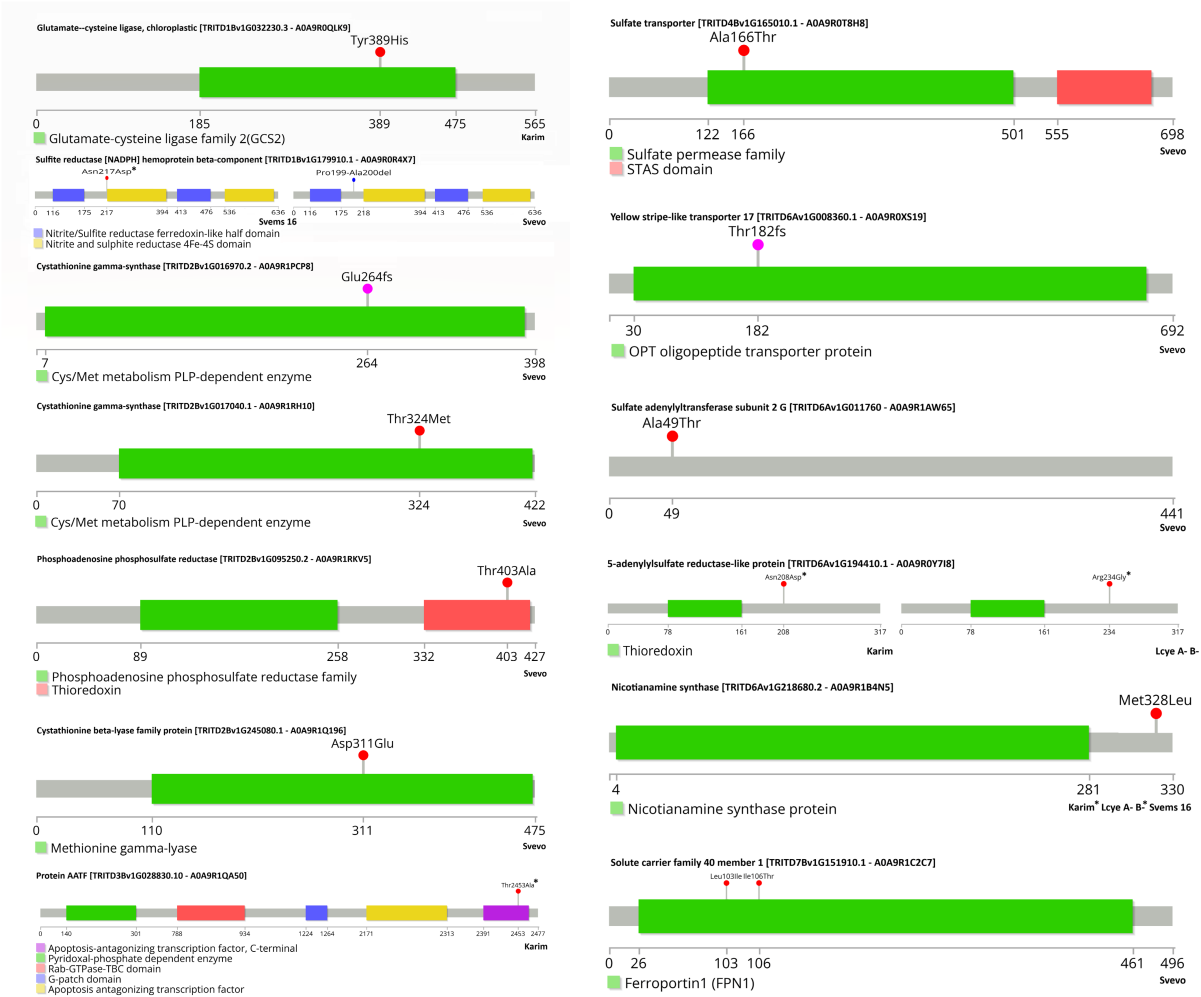
Lollipop plots of proteins encoded by genes putatively involved in S and/or Fe transport, metabolism, and homeostasis with high and moderate impact mutations on their coding sequence. For genes annotated in multiple isoforms, the data represented refer to the protein encoded by the Ensemble Canonical transcript. The asterisk (*) indicates homozygous mutations or genotypes that have a homozygous mutated allele.

To gain deeper insight into genetic differences potentially affecting S and/or Fe transport, metabolism, and homeostasis, we identified genes carrying moderate‐ or high‐impact coding variants (Figure 12). Svevo showed the highest number of affected genes (9 out of 13), followed by Karim (3), while LcyE A^−^B^−^ and Svems16 each harbored only two such variants. Most variants identified were missense mutations; however, Svevo also carried unique alterations such as frame‐shift and in‐frame deletions. Notably, Svevo harbored a frame‐shift mutation in the cystathionine‐gamma synthase gene (TRITD2Bv1G016970), caused by a G deletion at position 790, which disrupted the reading frame from glutamate at position 264 within a conserved PLP‐dependent Cys/Met metabolism domain (PF01053). Moreover, in Svevo, a gene encoding a yellow stripe‐like transporter 17 (TRITD6Av1G008360) had a C deleted at position 543, which altered the amino acid sequence from threonine at position 182, located within an OPT oligopeptide transporter protein family sequence (PF03169). Furthermore, this cultivar contained a six‐nucleotide conservative in‐frame deletion of the CCTGCA bases at positions 595 to 600 of the coding sequence of a gene whose product is the beta‐component of a sulfite reductase [NADPH] hemoprotein (TRITD1Bv1G179910); this led to the deletion of proline and alanine at positions 199 and 200, respectively. This same sulfite reductase gene also had a variant in Svems16, where an A at position 649 was substituted by a G, resulting in the switching of asparagine at position 217 with an aspartate, immediately upstream of a nitrite and sulfite reductase 4Fe‐4S domain (PF01077); Svems16 was homozygous for this variant.

Several missense variants were detected on other S metabolism genes. A gene encoding a chloroplastic glutamate‐cysteine ligase (TRITD1Bv1G032230) carried a T to C substitution at position 1165 in Karim, leading to the replacement of tyrosine at position 389 with a histidine within a glutamate‐cysteine ligase family 2 (GCS2) sequence (PF04107). Another cystathionine‐gamma synthase encoding gene (TRITD2Bv1G017040) characterized by a variant was found in Svevo, in which a C at position 971 mutated into a T, resulting in the change of threonine at position 324 to a methionine inside a cysteine/methionine metabolism PLP‐dependent enzyme domain (PF01053). Svevo also had a missense variant affecting an A at position 1207 of TRITD2Bv1G095250, whose encoded product is a phosphoadenosine phosphosulfate reductase; the substitution of this nucleotide with a G resulted in the change of threonine at position 403 inside a thioredoxin domain (PF00085) to an alanine. This cultivar additionally showed a T to A variant at position 933 of a cystathionine beta‐lyase family protein encoding gene (TRITD2Bv1G245080), which resulted in the replacement of aspartate by glutamate at position 311 within a methionine gamma‐lyase domain (PF06838).

A gene encoding a sulfate transporter (TRITD4Bv1G165010) had a missense variant involving a G at position 496 that turned into an A in Svevo, thereby replacing alanine at position 166 with a threonine in the first portion of a sulfate permease family sequence (PF00916). Svevo also carried a variant that affected a G at position 145 that changed into an A in TRITD6Av1G011760, whose encoded product, a sulfate adenylyltransferase subunit 2 G, had a substitution of alanine at position 49 with a threonine. The 5′‐adenylylsulfate reductase‐like protein encoding gene TRITD6Av1G194410 showed homozygous variants in both Karim and LcyE A^−^B^−^; the former genotype had a G instead of an A at position 622, which resulted in an aspartate at position 208 instead of asparagine, while the latter had a G at position 700 instead of an A, resulting in the substitution of arginine at position 234 with a glycine.

Regarding Fe transport or chelation genes, two variants very close to each other were found in Svevo on the coding sequence of TRITD7Bv1G151910, a gene encoding a solute carrier family 40 member 1. The T at position 307 changed into an A, leading to the substitution of leucine at position 103 with an isoleucine, while the replacement of a T at position 317 by a C resulted in the substitution of isoleucine at position 106 with a threonine. Both these variants were part of the ferroportin 1 (FPN1, PF06963) domain. Additionally, Karim, LcyE A^−^B^−^, and Svems16 shared the same variant in the TRITD6Av1G218680 gene, whose product is a nicotianamine synthase. Specifically, an A at position 982 switched to a C, leading to a leucine at position 328 instead of a methionine; this variant was homozygous in both Karim and LcyE A^−^B^−^.

A notable example is TRITD3Bv1G028830, initially annotated as an apoptosis‐antagonizing transcription factor (AATF) with a putative role in cysteine biosynthesis (GO:0006535; GO:00041). In Karim, this gene carried a homozygous A>G variant at position 7357, resulting in a threonine‐to‐alanine substitution at position 2453. However, BLAST analysis of the affected C‐terminal domain (PF08164) across Poaceae genomes suggested a likely misannotation, with this locus representing a fusion of multiple isoforms. One of these isoforms contains a pyridoxal‐phosphate‐dependent enzyme domain (PF00291), which better supports its involvement in cysteine metabolism.

## 3 Discussion

Fe deficiency significantly affects global agriculture, diminishing crop yields and quality, a critical concern given the need to feed a growing population (MacDonald 2016; Poole et al. 2021). This issue is compounded by the intricate relationship between Fe and S in plants (Astolfi et al. 2021). While S deficiency hinders Fe uptake, Fe deficiency can stimulate sulfate absorption (Astolfi et al. 2021). Interestingly, some research indicates that increased sulfate levels can boost Fe accumulation in wheat shoots under Fe‐deficient conditions, potentially through the production of PS, compounds facilitating Fe chelation and transport (Astolfi, Cesco, et al. 2006; Astolfi, Zuchi, et al. 2006; Zuchi et al. 2012). However, this positive effect of S is not consistent across all grasses, as barley and maize show different responses, underscoring the complexity and species‐specific nature of Fe–S interactions (Celletti, Paolacci, et al. 2016). To explore this variability, we assessed how four genetically distinct durum wheat genotypes respond to supra‐optimal S availability in terms of Fe accumulation. The panel included two widely cultivated varieties, Svevo (Italian) and Karim (Tunisian), and two promising drought‐tolerant genotypes, LcyE A^−^B^−^ and Svems16 (Quagliata et al. 2023), selected for their relevance in breeding climate‐resilient wheat adapted to Mediterranean conditions.

Fe deficiency stunts plant growth, reducing shoot and root biomass (Wang et al. 2022) and causing leaf chlorosis (Marschner 1995). Our findings revealed that both genotype and Fe/S availability significantly affected biomass development (Table S1). The absence of a significant genotype‐environment interaction (Table S1), however, indicates that the genotypes generally exhibited similar trends in biomass response to varying Fe and S levels, despite differences in their absolute growth. Notably, Fe deficiency reduced root biomass only in Svems16, a negative effect reversed by high S availability in Fe‐deficient plants. This finding suggests that S may enhance Fe uptake or alleviate Fe deficiency impacts specifically in Svems16 roots. Furthermore, Karim showed pronounced chlorosis under Fe deficiency, which was completely reversed by high S in Fe‐deficient plants (Figure 2c). This strongly implies a protective role of S, potentially through its involvement in chlorophyll precursor synthesis (Sharma et al. 2024) or enhanced Fe mobility within the plant, as S is also crucial for Fe translocation (Wu et al. 2020).

Root morphology controls soil resource acquisition efficiency (Lynch et al. 2022). The analysis of root architecture revealed that each genotype showed a specific root architecture differentially affected by growth conditions and their interactions (Table S1). High S conditions (ES, ESF) generally clustered together for most genotypes, as did control (C) and Fe‐deficient (F) conditions, except for Svevo, where C, ES, and ESF clustered together, suggesting Svevo's root system under ESF resembled that under sufficient Fe (Figure 3). The reduced root development observed under Fe deficiency (Figures 3 and S1) corroborates prior findings (Gruber et al. 2013). Notably, Svevo and LcyE A^−^B^−^ displayed the most substantial root growth reduction in Fe‐deficient conditions, indicating their greater sensitivity to low Fe availability compared to Karim and Svems16. This stunted growth could arise from impaired cell division linked to altered gene expression (Hua et al. 2022) and a strategic growth constraint to conserve limited nutrients (Seguela et al. 2008). Intriguingly, combining Fe deficiency with high S (ESF) significantly recovered root development in most genotypes. This finding suggests that S can partially offset the negative impacts of Fe deficiency on root development, possibly by supporting metabolic processes like amino acid and protein synthesis crucial for cell growth, or by influencing root‐related plant hormones (Lucena et al. 2015). However, Svevo's root volume and Karim's root tip number did not recover under combined Fe deficiency and high S (Figure S1), highlighting genotype‐specific responses and suggesting differing regulatory mechanisms in these genotypes. By analyzing the Fe concentration in plant tissues (Figure 6a,b), a general reduction of Fe concentration in both roots and shoots under Fe deficiency was found consistently with expectations, with Karim being the most affected, suggesting a potentially less efficient mechanism for Fe uptake or internal distribution compared to the other genotypes examined. However, the differential responses to supra‐optimal S levels revealed intricate regulatory mechanisms at play. Under high S conditions, Svevo showed decreased root Fe, possibly due to downregulated uptake as a protective mechanism against imbalance or toxicity. In contrast, Svems16 exhibited increased root Fe, potentially due to stimulated uptake linked to a higher demand for Fe‐S proteins or a mechanism to tolerate high root Fe when S is abundant. These contrasting responses highlighted different regulatory strategies influenced by each genotype's genetic background. Notably, LcyE A^−^B^−^ exhibited a distinctive behavior under high S alone (ES), showing a significant increase in shoot Fe. This response in this drought‐tolerant genotype may be linked to its adaptation to stress conditions, where prioritizing efficient nutrient uptake is often critical (Quagliata, Molina, et al. 2025). Supporting this idea, LcyE A^−^B^−^ also showed the highest PS release rate under Fe deficiency alone (F) (Figure 6c), suggesting an inherent capacity for efficient Fe acquisition. This capability might be intrinsically linked to its drought tolerance, as effective nutrient uptake mechanisms are frequently essential for survival and growth under water‐limited conditions (Maghrebi et al. 2024; Quagliata, Molina, et al. 2025).

The remarkable increase in shoot Fe concentration in Karim under combined Fe deficiency and high S (ESF) is particularly noteworthy. This suggests that even in a Fe‐deficiency‐susceptible genotype, an extra S supply can significantly enhance Fe acquisition or its transport to the shoots when Fe is limited, confirming our previous finding (Zuchi et al. 2012). This beneficial effect of S could be linked to enhanced production of PS or more efficient Fe loading into the xylem. Actually, in line with what was reported by Zuchi et al. (2012), the ESF condition stimulated the PS release rate in Svevo plants, supporting the hypothesis of a direct link between S supply and PS biosynthesis (Astolfi, Cesco, et al. 2006; Astolfi, Zuchi, et al. 2006). Since PS are derived from methionine (Kobayashi and Nishizawa 2012), increased S availability could fuel the production of PS, thereby enhancing Fe uptake. The fact that Fe deficiency upregulates the Yang cycle, which recycles methionine for PS synthesis (Lucena et al. 2015), further strengthens this connection. Furthermore, this finding indicates that the S effect on PS production or release is likely genotype‐dependent and not only species‐specific, as previously demonstrated (Celletti, Paolacci, et al. 2016). The observed differences in Fe accumulation and utilization among these genotypes could be explained by specific variants in Fe‐related genes. For example, the Solute Carrier Family 40 member 1 gene (TRITD7Bv1G151910), involved in Fe transport, carries a missense mutation in LcyE A^−^B^−^, Svems16, and Karim compared to Svevo, which may alter protein function and impact Fe uptake or distribution. Furthermore, a second copy of this gene, TRITD4Bv1G185470, harbors a downstream variant in LcyE A^−^B^−^ and Svems16, potentially affecting its post‐transcriptional regulation and contributing to genotype‐specific differences in Fe homeostasis.

To further investigate the potential link between S availability and PS production/release suggested by the previous findings, we analyzed the total S concentration in plant tissues. The significant interaction effect observed in the ANOVA (Table S1) underscored the genotype‐specific nature of S accumulation under different growth conditions. Svevo consistently exhibited low S concentrations in both shoots and roots, a trait unaffected by any of the tested conditions. This generally low S translocation rate in Svevo could be attributed to genetic variations in sulfate transporter encoding genes. Specifically, the gene TRITD4Bv1G165010, which encodes a sulfate transporter, contains a missense variant in this genotype, leading to the replacement of an alanine with the polar amino acid threonine within its sulfate permease family sequence (PF00916) (Figure 12), as previously reported by Quagliata, Molina, et al. (2025). The generally stable S concentrations in the ES condition compared to the control (C) aligns with the understanding of sulfate uptake regulation (Davidian and Kopriva 2010). The downregulation of sulfate transporters when S supply is abundant, or even adequate (Hawkesford 2003), explains why most genotypes did not increase S accumulation in their tissues under these conditions. However, the exceptions of Svems16, showing decreased S accumulation, and LcyE A^−^B^−^, showing increased S accumulation, suggest unique regulatory mechanisms or metabolic demands in these genotypes. On the other hand, the elevated root S accumulation in Karim under ESF provides compelling evidence for an increased S demand when the plant is struggling with Fe acquisition. This aligns perfectly with the hypothesis that S is likely being channeled towards PS biosynthesis, occurring specifically in the roots where Met is synthesized de novo, rather than translocated from the epigeal tissues (Nakanishi et al. 1999). In this context, the shoot could act as the primary site of sulfate assimilation (Ravanel et al. 2004), and the resulting S‐containing metabolites (mainly thiols) are then mobilized to the roots as S donors for the synthesis of Met to fuel the production of PS and thereby enhance Fe uptake. Interestingly, our analysis revealed specific genetic variations in genes putatively involved in methionine metabolism and PS biosynthesis and transport (Figures 11 and 12). Several mutations affecting methionine metabolism genes were identified in Svevo, all located on chromosome 2B and within conserved domains essential for enzymatic activity. These alterations suggest a disrupted methionine biosynthetic or catabolic pathway in this genotype, potentially limiting precursor availability for PS production. Moreover, variants were found in two genes likely involved in PS biosynthesis and transport. One encodes a nicotianamine synthase, a key enzyme initiating the mugineic acid pathway (Bashir and Nishizawa 2006) and harbored a shared variant across three genotypes. The other, a yellow stripe‐like transporter involved in metal‐PS complex trafficking (Islam et al. 2020), showed a severe structural alteration in Svevo, possibly compromising Fe chelate export. Together, these genomic features may contribute to Svevo's limited capacity to mobilize Fe under stress conditions.

Overall, these findings connected S availability, root‐localized PS biosynthesis, and the consequent enhancement of Fe acquisition, particularly in a genotype susceptible to Fe deficiency like Karim. The hypothesis that thiol compounds could play a crucial role in the plant response to Fe stress, likely by providing the necessary precursors for PS synthesis in the roots, seems to be supported by the consistent increase in root thiol concentration under Fe deficiency across almost all genotypes (Figure 4c). The particularly marked increase in Svems16 and Karim aligns with Karim's observed enhanced PS release under Fe deficiency, suggesting a coordinated effort to mobilize S resources in the roots of these genotypes to facilitate Fe acquisition. On the other hand, the relative stability of shoot thiol concentration across most treatments suggests that the shoot total thiol pool is less directly responsive to fluctuating Fe and S availability compared to the roots. The variable responses to the combined Fe deficiency and high S (ESF) condition are particularly intriguing. The increase in root thiols in Svevo and Svems16 under ESF compared to ES (Figure 4c) suggests that when both high S and Fe deficiency signals are present, these genotypes might further upregulate thiol accumulation in the roots. A key candidate gene implicated in thiol synthesis is TRITD1Bv1G032230, putatively encoding a chloroplastic glutamate‐cysteine ligase. This enzyme is crucial as it catalyzes the rate‐limiting step in glutathione (GSH) biosynthesis. Interestingly, in Karim, this gene carries a missense mutation within its conserved glutamate‐cysteine ligase family 2 (GCS2) domain (PF04107), potentially influencing its activity or regulation (Figure 12). While the role of GSH in Fe tolerance and accumulation has been established (Shanmugam et al. 2015; Rajab et al. 2020), a recent report demonstrated that Fe sensitivity is not solely determined by total GSH levels (Khan et al. 2022). The bottlenecks in sulfite reduction and/or GSH biosynthesis differentially regulate the reciprocal signaling between Fe and S networks, as well as the expression of Fe homeostasis, S uptake/transport, and Fe–S biogenesis marker genes (Khan et al. 2022). However, the lower thiol accumulation in both tissues of Svems16 and roots of Karim under ESF compared to Fe deficiency alone (F) points towards a potential inhibitory effect of S excess on thiol accumulation when Fe is limiting in these genotypes. This could indicate a complex feedback regulation where excessive S might, in some cases, hinder the plant internal signaling pathways related to Fe acquisition, potentially by altering the demand for PS precursors (Kopriva et al. 2016).

The detailed analysis of ATPS and OASTL activity, the first and last enzyme in the assimilation pathway, respectively (Leustek et al. 2000; Kopriva 2006; Krishnan et al. 2018), provided a crucial mechanistic link between the discussed dynamics of total S and thiol accumulation and the plant response to Fe deficiency, further corroborating the genotype‐specific differences observed in the previous sections. In general, higher shoot ATPS activity in Svevo and Karim under control conditions (Figure 5b) aligns with the established role of leaves as the primary site of sulfate assimilation (Lewandowska and Sirko 2008), whereas a more balanced root/shoot ATPS activity in Svems16 and LcyE A^−^B^−^ suggests a different allocation strategy of S assimilation in these genotypes even without stress. The significant increase in root ATPS activity in roots of Svevo and Karim under Fe deficiency (Figure 5a), coupled with the corresponding rise in root thiol concentration (Figure 4c), strongly supports the hypothesis that these genotypes actively upregulate the initial steps of S assimilation in the roots when faced with Fe limitation. This localized increase in S assimilation rate likely serves to provide the necessary precursors for PS biosynthesis in the roots, where these chelators are released into the rhizosphere to enhance Fe uptake. The reduced shoot ATPS found in LcyE A^−^B^−^ under Fe deficiency (Figure 5b) might indicate a different strategy, prioritizing root metabolism at the expense of shoot S assimilation. The variable ATPS responses to high S alone (ES) across genotypes underscore the complex regulatory network governing S metabolism, which is not solely dictated by S availability but is also intricately linked to the plant genetic background (Table S1). The inhibition of root ATPS activity in Svems16 under ES (Figure 5a) is consistent with other studies in the literature (Lappartient and Touraine 1996; Zuchi et al. 2012). The pattern of ATPS activity under combined Fe deficiency and high S (ESF) was even more complex. The reduction in root ATPS activity in Svevo and Svems16 under ESF compared to F (Figure 5a) suggests a potential feedback mechanism where excessive S, in the context of Fe deficiency, might downregulate the initial steps of sulfate assimilation in these genotypes. Conversely, the increased ATPS activity in roots of Karim and LcyE A^−^B^−^ and in shoots of Svevo and LcyE A^−^B^−^ (Figure 5a,b) under ESF indicates a different regulatory strategy, possibly aimed at maximizing S assimilation to support PS production when Fe was present at non‐optimal levels. To further pinpoint potential metabolic bottlenecks contributing to differential Fe deficiency responses, we examined OASTL activity (Figure 5c,d). The consistently low OASTL activity in Karim provides a potential biochemical explanation for its higher susceptibility to Fe deficiency. A reduced capacity to synthesize cysteine, a key intermediate in both general S metabolism and the precursor for methionine and PS, could limit its ability to mount an effective response to Fe stress. The differential OASTL responses to Fe deficiency further underscore the genotype‐specific strategies for coping with Fe stress, with some genotypes, such as LcyE A^−^B^−^ and Karim, upregulating cysteine synthesis in roots, and others, like Svems16 and LcyE A^−^B^−^, in shoots, likely to support enhanced PS production. Finally, the increased OASTL activity in shoots and roots of Svevo and roots of Svems16 under ESF compared to F suggests a targeted response to the combined stress, which was previously described by Zuchi et al. (2012). In particular, the enhanced cysteine synthesis in the root tissues of these genotypes could indeed contribute to mitigating the effects of Fe deficiency by ensuring a sufficient supply of precursors for PS production in the primary site of Fe acquisition. The impact of Fe deficiency extends beyond the S assimilation pathway, leading to altered ionomic profiles in plant tissues by affecting the uptake of other nutrients (Baxter et al. 2008). Indeed, our analysis of root tissue ionomics under single and combined Fe deficiency (F and ESF conditions) revealed significant changes, with the modulation of nutrient accumulation varying considerably among the different genotypes (Figure 7). The general clustering of Fe‐deficient conditions (F and ESF) together, distinct from the control (C) and excess S (ES) treatments for most genotypes, strongly suggests that Fe deficiency is the primary driver of altered nutrient accumulation in the roots. This implies that the plant's homeostatic mechanisms are significantly perturbed by the lack of Fe, triggering a suite of changes in the uptake and accumulation of other elements (Hua et al. 2022; Quagliata, Ferrucci, et al. 2025), regardless of whether excess S is also present. However, the unique clustering pattern observed in LcyE A^−^B^−^, where control (C), Fe deficiency (F), and excess S (ES) grouped together, with ESF condition standing alone, indicates a distinct regulatory response in this genotype and suggests that S excess somehow alters the impact of Fe deficiency within this genotype. Additionally, the mineral elements themselves formed distinct clusters based on how their concentrations correlate with the different S and Fe supply, highlighting potential co‐regulation or similar uptake/transport mechanisms among certain mineral nutrients within each genotype under the tested conditions. For instance, the group comprising Zn and Cu exhibited similar trends in most genotypes, as did another group consisting of Ca and Mg (Figure 7). In particular, Fe deficiency led to an increase in Cu accumulation in plant roots in all genotypes, except LcyE A^−^B^−^, likely a compensatory mechanism for reduced Fe availability in redox and enzymatic functions (Printz et al. 2016). Consistent with findings in tobacco and Arabidopsis (Kobayashi et al. 2003; Thomine et al. 2003), Zn accumulation occurred under Fe deficiency across all genotypes. This is likely due to the dual transport capability of IRT1 and IRT2 proteins for both Fe and Zn, as demonstrated in Arabidopsis (Vert et al. 2001; Henriques et al. 2002) and barley (Pedas et al. 2008). In grasses, the broad specificity of YS1 (Kanai et al. 2009) might also contribute to this phenomenon. On the other hand, excess S alone (ES) generally led to a decrease in most nutrient concentrations. The more intricate clustering patterns observed in shoot tissues compared to roots suggest that nutrient translocation and accumulation in the aerial parts of the plant are governed by a more complex interplay of factors beyond just the external root environment. For instance, in both Svevo and Karim, C and F conditions clustered together while F and ESF stood alone. Svevo's shoots under ESF showed a general decrease in most nutrients but an increase in Zn and Mg, suggesting a specific reallocation strategy under combined stress. In Svems16 shoots, the control condition clustered alone, and Fe deficiency, excess S, and their combination elicited similar alterations, with ESF leading to an overall increase in nutrient accumulation. Shoot tissues of LcyE A^−^B^−^ showed similar responses to F and ESF, characterized by decreased accumulation of most nutrients but increased Mg and Zn, highlighting Fe deficiency as a key driver in this genotype's shoot ionomics. Finally, Karim's shoots showed ES clustered distinctly, with decreased Mn and Ca and increased K, while F and ESF clustered with the control, suggesting a less pronounced impact of Fe deficiency on the overall shoot ionome of this genotype under these experimental conditions.

To investigate whether the differential accumulation of Fe and other nutrients observed in the root and shoot tissues of our tested genotypes translates to the grain, we conducted a follow‐up experiment under greenhouse conditions. Overall, the interaction between Fe deficiency and excess S can have direct agronomic consequences, with Svems16 showing a potential yield advantage and improved plant health under the combined treatment (Table 1). The higher chlorophyll content in this genotype under ESF (Table 1) also hints at a possible link to enhanced grain nutritional quality, although direct analysis of grain nutrient composition did not confirm this (Figures 9 and S2). The analysis of grain Fe accumulation further confirmed the higher susceptibility of Karim to Fe deficiency, evidenced by the significant reduction in grain Fe accumulation under Fe‐limiting conditions (Figure 8). The greater resilience observed in other genotypes, where grain Fe levels remained comparable to control plants despite Fe deficiency, highlighted the inherent genetic variability in Fe‐use efficiency among durum wheat cultivars (Coppa et al. 2023, 2025). The suggested role of adequate or supra‐optimal S supply in mitigating the negative impacts of Fe deficiency and even positively influencing Fe accumulation in grains (Zuchi et al. 2012; Astolfi et al. 2018) is further substantiated by our findings. The enhanced Fe accumulation in the grains of LcyE A^−^B^−^ under combined Fe deficiency and excess sulfate (ESF) is a particularly significant observation (Figure 8). Remarkably, this occurred despite low Fe concentrations in the root and shoot tissues of this genotype under the same conditions, suggesting a highly efficient Fe translocation and remobilization mechanism to the grain, specifically promoted by the supra‐optimal S availability. The positive impact of excess sulfate (ES) on grain Fe accumulation in Svems16 further supports the beneficial role of S in enhancing Fe uptake, translocation, and ultimately, accumulation in the grain of this genotype. This trait in durum wheat represents a high‐priority target for biofortification breeding programs, as it enables improved grain quality under micronutrient‐limited environments. This could align with the known effect of S deficiency, which instead impairs the plant's ability to activate its Fe uptake machinery under Fe‐limiting conditions (Astolfi et al. 2021). Consequently, when S is scarce and Fe is deficient, the plant's capacity to absorb not only Fe but also other divalent cations like potentially toxic Mn and Zn is limited (Robe et al. 2020). On the other hand, the lack of a positive response to excess sulfate in Karim and Svevo, even under Fe‐sufficient conditions, indicates that the beneficial interaction between S and Fe accumulation in the grain is not a shared response but rather a genotype‐specific response. As discussed for root and shoot tissues, the availability of Fe and S in the growth medium significantly influenced the accumulation of other macro‐ and micronutrients in the grain across the tested genotypes. The most prominent observation is the distinct elemental concentration patterns across the four genotypes under the same experimental conditions. This strengthens the significant genetic control over grain mineral composition. For instance, in the Svevo genotype (Figure 9), the F (Fe deficiency) condition clustered distinctly from the closely grouped C (Control), ES, and ESF conditions. This clustering pattern indicates that the overall elemental concentration profile in the grain under control, high sulfate, and combined stress was more similar to each other than to that observed under Fe deficiency. Consistent with this, Astolfi et al. (2018) previously reported phenotypic differences in Svevo grains from Fe‐deficient plants, using μ‐XRF imaging to detail Fe distribution. Their findings revealed that Fe‐deficient grains were smaller than those from the other conditions and exhibited a different spatial distribution of Fe within the grain. These observations suggested that Fe deficiency in Svevo not only reduces Fe concentration but also has broader consequences for Fe localization and the overall physical development of the grain, potentially impacting yield and quality. In Svems16, ES condition clustered alone (Figure 9), likely indicating that supra‐optimal sulfate had a relatively major impact on the overall mineral profile compared to Fe deficiency in this genotype. Finally, the clustering pattern for both LcyE A^−^B^−^ and Karim genotypes was similar, with ESF condition clustering alone (Figure 9). Furthermore, the elemental clustering patterns revealed genotype‐specific relationships. In both Svems16 and Karim, zinc (Zn) and manganese (Mn) clustered closely (Figure 9), suggesting a similar response in their relative accumulation within the grain across the different experimental conditions in these two genotypes. This close association could indicate shared uptake or transport mechanisms or similar regulatory pathways influencing their accumulation (Rai et al. 2021). Conversely, in Svevo and LcyE A^−^B^−^, Ca and Mo exhibited close clustering (Figure 9). This suggests a distinct relationship between the accumulation of these two elements in these specific genotypes under varying S and Fe availability. This could point towards different physiological mechanisms at play in Svevo and LcyE A^−^B^−^ compared to Svems16 and Karim regarding the uptake, translocation, or storage of Ca and Mo. Notably, Mo accumulation under Fe deficiency has been previously reported (Yang et al. 2010), and our findings corroborate this in three out of four genotypes (Svevo, LcyE A^−^B^−^ and Karim) (Figures 9 and S2). This suggests a potential systemic response to Fe stress that influences Mo uptake or translocation in these genotypes. The genetic basis of Mo accumulation in wheat grains is further supported by a genome‐wide association study (GWAS) by Jin et al. (2022), which identified significant single‐nucleotide polymorphisms (SNPs), particularly on chromosome 2A, associated with Mo content. Candidate genes identified include molybdate transporters and a molybdopterin biosynthesis protein, highlighting the specific genetic machinery influencing Mo accumulation and potentially its relationship with other elements like Ca under Fe deficiency in genotypes like Svevo and LcyE A^−^B^−^. The close clustering of Ca and Mo in these genotypes might therefore reflect a complex interplay between the physiological response to Fe deficiency, the genetically determined capacity for Mo accumulation, and potentially shared or linked pathways affecting Ca dynamics.

In conclusion, the observed S and Fe interaction in wheat is intricate and highly genotype‐dependent, underscoring the complexity and variability of nutrient interactions in plants. The LcyE A^−^B^−^ genotype demonstrated remarkable efficiency in Fe translocation and storage, effectively accumulating Fe in grains under combined excess S and Fe deficiency (ESF), thus despite low Fe supply. Conversely, Svems16 exhibited increased grain Fe accumulation under supra‐optimal S (ES) alone. Karim, however, proved to be the most sensitive to Fe deficiency, showing a limited response to supra‐optimal S and suggesting inefficiencies in Fe uptake and transport. The analysis of ATPS and OASTL enzymatic activity further confirmed the crucial role of S metabolism in the plant response to Fe deficiency, with increased activity likely indicating accelerated synthesis of thiol compounds essential for Fe uptake in Strategy II systems. Notably, the impact of supra‐optimal S on these enzymes was highly genotype‐specific (Table S1). Furthermore, the distinct nutrient clustering patterns across genotypes emphasized the complex and genotype‐specific nature of elemental interactions within durum wheat root and shoot tissues and grains under varying nutritional conditions.

Despite the inherent differences between controlled hydroponic and greenhouse environments, several genotype‐specific trends were conserved, particularly in terms of chlorophyll recovery and Fe accumulation under ESF. However, the genotype‐specific responses in yield traits and grain ionomics emphasize the need to validate these physiological responses under field‐like conditions to confirm their agronomic relevance.

## 4 Conclusion

This study introduces a novel, integrated strategy for durum wheat, combining drought tolerance with Fe biofortification. This dual approach directly addresses the urgent challenges of climate change and widespread micronutrient malnutrition.

Our systematic multilevel analyses revealed that S availability significantly promotes Fe homeostasis and enhances biofortification potential. We remarkably identified specific genotypes, such as LcyE A^−^B^−^, which demonstrated superior grain Fe accumulation even in Fe‐limited conditions. This suggests a potential synergistic interaction with S, offering a promising avenue for future biofortification breeding efforts.

The identification of genotype‐specific genetic differences underpinning these shoot Fe phenotypic variations in both major Fe functional pathways and S homeostasis pathways further reinforces the potential for targeted breeding programs. These programs could effectively capitalize on the synergistic interaction between Fe and S nutrition. While our current findings provide a robust phenotypic and genetic foundation, future studies incorporating gene expression analysis, such as through qRT‐PCR, would provide a more comprehensive understanding of the molecular mechanisms supporting the observed physiological and phenotypic responses. The genetic variants we identified in key genes involved in Fe transport, S translocation, and thiol synthesis are likely to have a functional impact. Thus, the functional characterization of these candidate genes will be crucial for validating their role in Fe and S homeostasis and leveraging this knowledge for molecular breeding. This integrated approach lays the groundwork for developing climate‐resilient, Fe‐enriched wheat varieties, a crucial step toward ensuring sustainable food security.

## 5 Materials and Methods

### 5.1 Hydroponic Experiment

This study utilized four diverse durum wheat ( *Triticum turgidum* ssp. *durum* ) genotypes: Svevo and Karim, representing commercial cultivars, and LcyE A^−^B^−^ and Svems16, selected for their demonstrated drought tolerance (Quagliata et al. 2023). LcyE A^−^B^−^ is a mutant line with double null homoalleles of the ε‐lycopene cyclase (LcyE) enzyme, which accumulates only β‐carotene, a Vitamin A precursor, in its caryopses (Sestili et al. 2019). Svems16 is a TILLING mutant line derived from Svevo (Bovina et al. 2014). The seeds were soaked in distilled water for 1 h and allowed to germinate in aeroponics in the dark at 28°C for 4 days. Homogeneous seedlings were selected and transferred to hydroponic culture, with six plants per pot containing 2.2 L of nutrient solution (NS) (Zhang et al. 1991). Plants were exposed to optimal and supra‐optimal sulfate (1.2 and 2.4 mM sulfate, respectively) and optimal and Fe‐deficient conditions (80 and 20 μM FeIII‐EDTA, respectively). Sulfate concentrations were selected based on previous reports (Zuchi et al. 2012; Celletti, Pii, et al. 2016), with 1.2 mM representing an optimal (C) and 2.4 mM a supra‐optimal (ES) supply. Similarly, 80 μM Fe (III)‐EDTA represented a sufficient and 20 μM a deficient Fe supply (Celletti, Paolacci, et al. 2016). NS was continuously aerated and changed every 3 days. The growth conditions were 27°C/20°C with 14/10 h day/night cycles and relative humidity of 80% and 200 mmol m^−2^ s^−1^ PAR at leaf level. Nine days after sowing, wheat shoots and roots were sampled. At harvest, four treatments were established: control (C, 1.2 mM sulfate/80 μM FeIII‐EDTA); Fe‐deficient (F, 1.2 mM sulfate/20 μM FeIII‐EDTA); Supra‐optimal S (ES, 2.4 mM sulfate/80 μM FeIII‐EDTA); Supra‐optimal S/Fe‐deficient (ESF, 2.4 mM sulfate/20 μM FeIII‐EDTA).

### 5.2 Greenhouse Experiment

Seeds of the same four durum wheat genotypes were germinated as previously described in Section 2.1. Germinated seedlings were then transferred to 20 cm diameter plastic pots (three seedlings per pot) containing a 3 L mixture of 50% (v/v) sand and perlite. Plants were grown in a low‐technology greenhouse, relying on natural ventilation through wall and roof windows and natural sunlight for photoperiod. Plants were watered with 1 L per pot of NS (Zhang et al. 1991) three times weekly (Monday, Wednesday, Friday), and 1 L per pot of demineralized water on alternate days (Tuesday, Thursday, Saturday). Pots were allowed to drain freely to prevent nutrient accumulation. Plants were exposed to optimal and supra‐optimal sulfate (1.2 and 2.4 mM sulfate, respectively) and optimal and Fe‐deficient conditions (80 and 20 μM FeIII‐EDTA, respectively), as described in paragraph 2.1. This resulted in four treatment conditions: C, F, ES, and ESF. NS was supplied until approximately 56 days post‐anthesis, after which plants were allowed to senesce without water until harvest at 170 days post‐sowing.

### 5.3 Chlorophyll Content

The chlorophyll content in leaves was estimated by the Soil Plant Analysis Development, a portable non‐destructive device (SPAD Meter—502 Plus, Konica Minolta Co.) using the youngest fully expanded leaf. Readings were expressed as SPAD units.

### 5.4 Root Morphological Analysis

At harvest, the root system architecture was evaluated using WinRhizo software (Regent Instruments Inc.) from images obtained by placing roots in a water‐filled plexiglass tray to minimize overlap. Measured parameters included length, surface area, volume, average diameter, and number of root tips.

### 5.5 Determination of Macro‐ and Micronutrient Concentrations

Macronutrient (Mg, Ca, K) and micronutrient (Fe, Mn, Cu, Zn, and Mo) concentrations were determined in oven‐dried (80°C for 48 h) shoot and root tissues. Samples were then mineralized using a microwave digestion system (Multiwave Go Plus, Anton Paar GmbH) with a mixture of ultrapure HNO_3_ 69.5% (3 mL) and HCl 37% (0.6 mL). Following mineralization and filtration, samples were diluted 1:200 with Milli‐Q water. Element concentrations were measured by inductively coupled plasma‐mass spectroscopy (Agilent ICP‐MS 7850).

### 5.6 Determination of Total S Concentration

Total S concentration was determined in oven‐dried (80°C for 48 h) shoot and root tissues. Dried samples were then ashed in a muffle furnace at 500°C for 24 h. The ashes were dissolved in 10 mL of HCl 3 N and filtered through Whatman No. 42 paper. The filtrate was reacted with 2% (w/v) BaCl_2_, forming a BaSO_4_ precipitate, which was determined turbidimetrically (Bardsley and Lancaster 1960).

### 5.7 Determination of Non‐Protein Thiol Concentration

The quantification of non‐protein thiol compounds was performed colorimetrically using 5,5′‐dithio‐bis‐(2‐nitrobenzoic acid) (DTNB), as described by Quagliata et al. (2023). In brief, frozen shoot and root fresh tissues (1 g FW) were homogenized in a solution containing 80 mM trichloroacetic acid (TCA), 1 mM ethylenediaminetetraacetic acid (EDTA), 0.15% (w/v) ascorbic acid (w/v), and 1% of polyvinylpolypyrrolidone (PVPP). After centrifugation at 6000× *g* at 4°C for 13 min, the supernatants were collected and the concentrations of DTNB‐reactive compounds were detected spectrophotometrically at 415 nm (Agilent Cary 3500 UV–Vis Spectrophotometer).

### 5.8 Extraction and Assay of ATPS and OASTL Activity

Frozen tissues were powdered with liquid N_2_. The extraction buffer (pH 7.4), containing 50 mM HEPES‐KOH, 5 mM MgCl_2_, 1 mM EDTA, 10% (v/v) glycerol, 0.1% (v/v) Triton x‐100, 5 mM dithiothreitol (DTT), 1 mM phenylmethylsulfonyl fluoride (PMSF), and 1% (w/v) polyvinylpyrrolidone (PVP), was added in a ratio of 1:3 and 1:7 (w/v) for root and shoot samples, respectively. The homogenate was filtered and centrifuged at 6000× *g* for 5 min at 2°C, and the supernatants obtained were centrifuged at 30,000× *g* for 5 min at 2°C and divided into aliquots that were frozen in liquid N_2_ and stored at −80°C until analysis. The activity of ATP sulfurylase (ATPS; EC 2.7.7.4) was determined by the bioluminescence technique according to Celletti, Pii, et al. (2016). The reaction mixture contained 8 μM APS, 16 mM tris‐acetate buffer pH 7.75, 40 μL ATP Monitoring Reagent, containing firefly luciferase (ThermoLabSystems), 68 μM Na_4_P_2_O_7_, and 30 or 20 μL of root or shoot crude extracts, respectively. Briefly, the light production catalyzed by firefly luciferase (EC 1.13.12.7) is coupled to the ATP production during the enzyme reaction, and the light emission was measured with a luminometer (LKB‐Wallace 1250). O‐acetylserine(thiol)lyase (OASTL; EC 4.2.99.8) activity was determined colorimetrically according to Celletti, Pii, et al. (2016), measuring the cysteine synthesis using ninhydrin. The crude extracts were added to a reaction mixture pH 8.0 (167 mM Tris–HCl and 16.7 mM DTT), 4 mM pyridoxal‐5′‐phosphate, and 250 mM OAS. The reaction was started by the addition of 50 mM Na_2_S, and after 10 min at 30°C, it was stopped at 100°C for 5 min after the addition of Gaitonde's reagent (0.25 g of ninhydrin in 16 mL of acetic acid and 4 mL of hydrochloric acid 37%), subsequently cooled to room temperature and diluted with 1 mL of ethanol. The values obtained spectrophotometrically at 560 nm (Agilent Cary 3500 UV–Vis) were related to a calibration line made with known concentrations of cysteine from 0 to 100 μL. The protein content in shoot and root extracts was determined by the protein‐binding Coomassie Brilliant Blue G‐250 dye method using bovine serum albumin as standard according to Bradford (1976).

### 5.9 Collection of Root Exudates and Determination of PS Release

The rate of PS release from wheat roots was quantified by determining PS content in root exudates. Wheat plants grown for 9 days were used. One hour after the start of the light cycle, the root systems were thoroughly washed in deionized water and then immersed in continuously aerated deionized water (one plant in 20 mL) for 3 h to collect released PS. The resulting solution was then processed by freeze‐drying (for 72 h, mainly at −56°C and with a pressure of 0.8 mbar), and the PS concentration was measured using the Fe‐binding assay according to Reichman and Parker (2006).

### 5.10 Genotyping‐by‐Sequencing (GBS) Analysis

#### 5.10.1 DNA Extraction

The genomic DNA was extracted from the four genotypes grown in a greenhouse under a 12‐h light/12‐h dark photoperiod and maintained at controlled temperatures of 24°C during the day and 16°C at night. Extractions were performed in duplicate from leaf tissue using the CTAB protocol (Murray and Thompson 1980). GBS library preparation, DNA quality control, enzymatic digestion, and sequencing were carried out by CD Genomics (45‐1 Ramsey Road, Shirley, NY, USA) using the NovaSeq platform. Each replicate yielded approximately 5 million PE150 reads per sample. The restriction enzymes PstI and MspI were used for DNA digestion.

#### 5.10.2 Bioinformatic Analysis

Following sequencing, raw reads were quality‐filtered and trimmed to remove adapters using fastp with the “detect_adapter_for_pe” option (Chen et al. 2018). Cleaned reads were aligned to the Svevo.v1 reference genome (Maccaferri et al. 2019) using BWA‐MEM (Li 2013). Subsequent quality control and duplicate marking were performed with samtools (Danecek et al. 2021). Variant calling was conducted using GATK HaplotypeCaller (Van der Auwera and O'Connor 2020). Single Nucleotide Polymorphisms (SNPs) and Insertions/Deletions (InDels) were subjected to stringent hard filtering based on the following thresholds: (i) SNPs: QD < 2.0, FS > 60.0, MQ < 40.0, SOR > 3.0, MQRankSum < −12.5, ReadPosRankSum < −8.0; (ii) InDels: QD < 2.0, FS > 200.0, SOR > 10.0, ReadPosRankSum < −20. Variant annotation was performed using SnpEff (Cingolani et al. 2012). To characterize the functional impact of genomic variants, Gene Ontology (GO) enrichment analysis was performed with the gprofiler2 package (Kolberg et al. 2020), using the gene sets affected per genotype. The P‐values were adjusted with the Benjamini–Hochberg procedure to control false positives, applying a column normalization of the −Log10(Padj‐value) for data visualization in the heatmap. Graphical representations were generated using ggplot2 (Wickham 2016) and complemented with pheatmap (Kolde and Kolde 2015). All computational analyses were carried out on the high‐performance computing (HPC) cluster of the Computing Center at the University of Córdoba (Córdoba, Spain), consisting of 24 Bull x440 nodes, each with 192 GB of RAM, totaling 960 processing cores.

### 5.11 Statistical Analysis

Each reported value represents the mean ± standard deviation (SD) of measurements performed in triplicate and obtained from three independent biological replicates. Data were analyzed using two‐way ANOVA to evaluate the effects of two independent factors on the dependent variables. The factors were the growth condition with four levels (C, F, ES, ESF) and the genotype with four levels (Svevo, Svems16, LcyE A^−^B^−^, Karim). The analysis was carried out using R software (Rstudio, V. 2023.12.0+369) and the packages: ggplot2 (version 3.5.1), dplyt (version 1.1.4), tidyverse (version 2.0.0), car (version 3.1‐3), and agricolae (version 1.3–7). To identify significant differences in dependent variables induced by the growth conditions within each genotype, one‐way ANOVA was followed by Tukey's post hoc tests (*p* < 0.05). The analysis and the plots were carried out using R software (Rstudio, V. 2023.12.0+369) and the packages: ggplot2 (version 3.5.1), dplyt (version 1.1.4), multcompView (version 0.1‐10), and ggpattern (version 1.1.4). Hierarchical clustering on root traits and ionomic datasets was performed with the full method and Euclidean distance measurement in R (V. 2023.12.0+369), using the packages pheatmap (version 1.0.12), RColorBrewer (version 1.1‐3), and gplots (version 3.1.3.1).

## Author Contributions

**Alessandro Bruschini:** writing – original draft, methodology, investigation, formal analysis, data curation. **Eleonora Coppa:** writing – original draft, formal analysis, data curation. **Giulia Quagliata:** methodology, investigation, formal analysis, data curation. **Miriam Marín‐Sanz:** writing – review and editing, formal analysis. **Andrea Ferrucci:** writing – review and edit, formal analysis, data curation. **Matteo Spada:** investigation. **Francesco Sestili:** writing – review and edit. **Francisco Barro:** writing – review and edit. **Gianpiero Vigani:** writing – review and editing. **Stefania Astolfi:** writing – original draft, writing – review and editing, supervision, funding acquisition, Conceptualization.

## Supporting information


**Figure S1:** Morphological parameters of the root system (length, surface area, volume, average diameter, number of tips) of four durum wheat genotypes grown hydroponically as described in Figure 1. Statistics as in Figure 2.
**Figure S2:** K, Mg, Ca, Mn, Cu, Zn, and Mo concentrations in root and shoot tissues of four durum wheat genotypes grown hydroponically and in grains of the same genotypes grown under greenhouse conditions as described in Figure 1. Statistics as in Figure 2.
**Table S1:** Two‐way ANOVA on physiological, morphological, and biochemical traits evaluated in four genotypes (Svevo, Svems16, LcyE A^−^B^−^, and Karim) grown in hydroponics.
**Table S2:** Two‐way ANOVA on agronomic traits (thousand‐seed weight, number of spikes per plant, spike weight, and chlorophyll content 22, 36, 49, and 63 days after transplanting—DAT) and nutrient accumulation (Fe, Cu, Zn, Mo, Ca, K, Mg, and Mn) evaluated in four genotypes (Svevo, Svems16, LcyE A^−^B^−^, and Karim) grown in greenhouse under different nutrient availability conditions: C, control; F, Fe‐deficiency; ES, supra‐optimal S; ESF, supra‐optimal S/Fe‐deficiency.

## Data Availability

The sequencing data analyzed during the current study is available from the corresponding author upon reasonable request.
