# Supplementary material for: Unveiling the Genetic and Physiological Synergies of Iron and Sulfur Homeostasis in Durum Wheat: From Root to Grain
Source: Physiol Plant. 2025 Sep 18;177(5):e70524. doi: 10.1111/ppl.70524 (PMC12446894; doi:10.1111/ppl.70524)
Supplement: Supplementary file 1 — Figure S1: Morphological parameters of the root system (length, surface area, volume, average diameter, number of tips) of four durum wheat genotypes grown hydroponically as described in Figure 1. Statistics as in Figure 2. Figure S2: K, Mg, Ca, Mn, Cu, Zn, and Mo concentrations in root and shoot tissues of four durum wheat genotypes grown hydroponically and in grains of the same genotypes grown under greenhouse conditions as described in Figure 1. Statistics as in Figure 2. Table S1: Two‐way ANOVA on physiological, morphological, and biochemical traits evaluated in four genotypes (Svevo, Svems16, LcyE A−B−, and Karim) grown in hydroponics. Table S2: Two‐way ANOVA on agronomic traits (thousand‐seed weight, number of spikes per plant, spike weight, and chlorophyll content 22, 36, 49, and 63 days after transplanting—DAT) and nutrient accumulation (Fe, Cu, Zn, Mo, Ca, K, Mg, and Mn) evaluated in four genotypes (Svevo, Svems16, LcyE A−B−, and Karim) grown in greenhouse under different nutrient availability conditions: C, control; F, Fe‐deficiency; ES, supra‐optimal S; ESF, supra‐optimal S/Fe‐deficiency. [file PPL-177-e70524-s001.pdf]

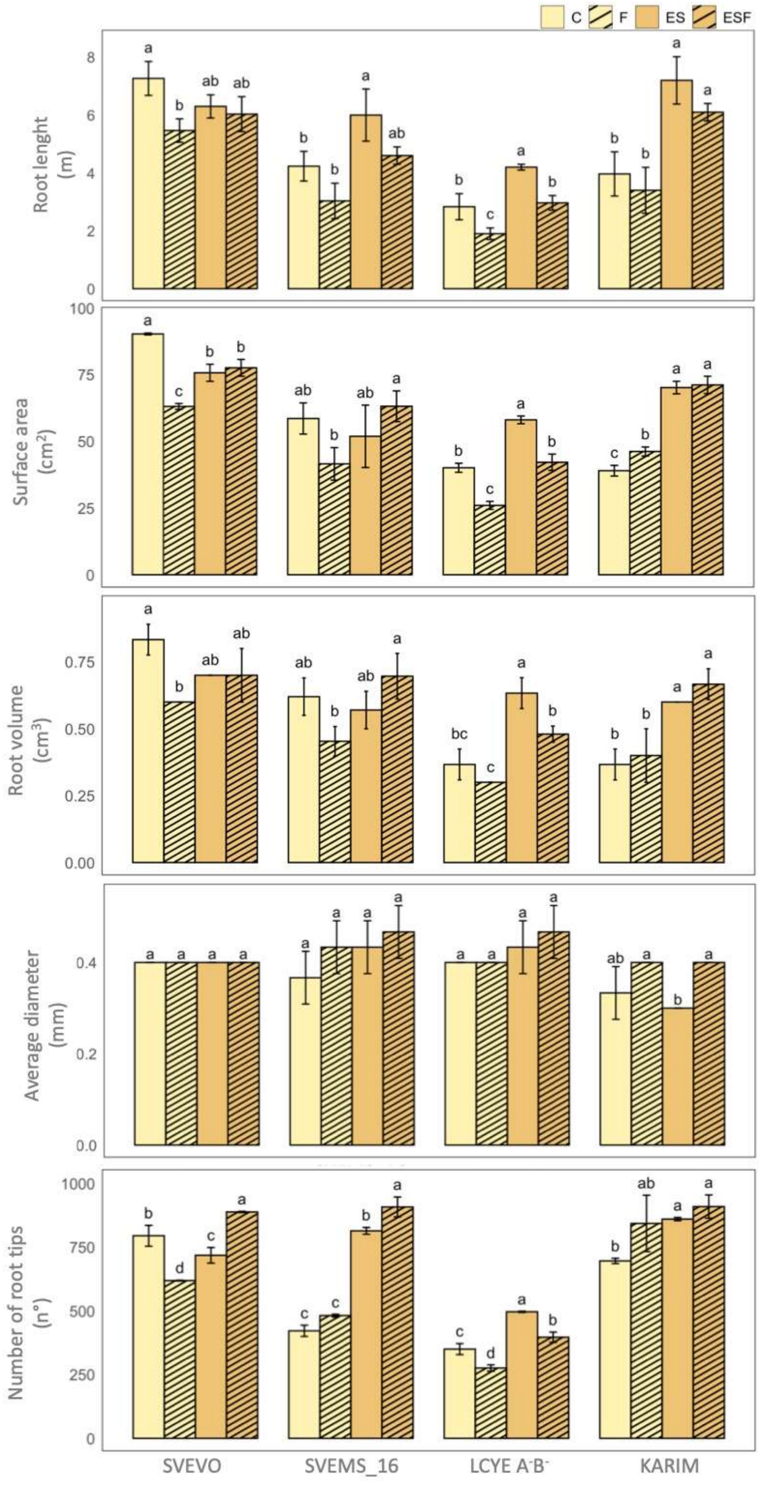

Supplementary Figure 1 (Fig. S1). Morphological parameters of root system (length, surface area, volume, average diameter, number of tips) of four durum wheat genotypes grown hydroponically as described in Fig.1. Statistics as in Figure 2.

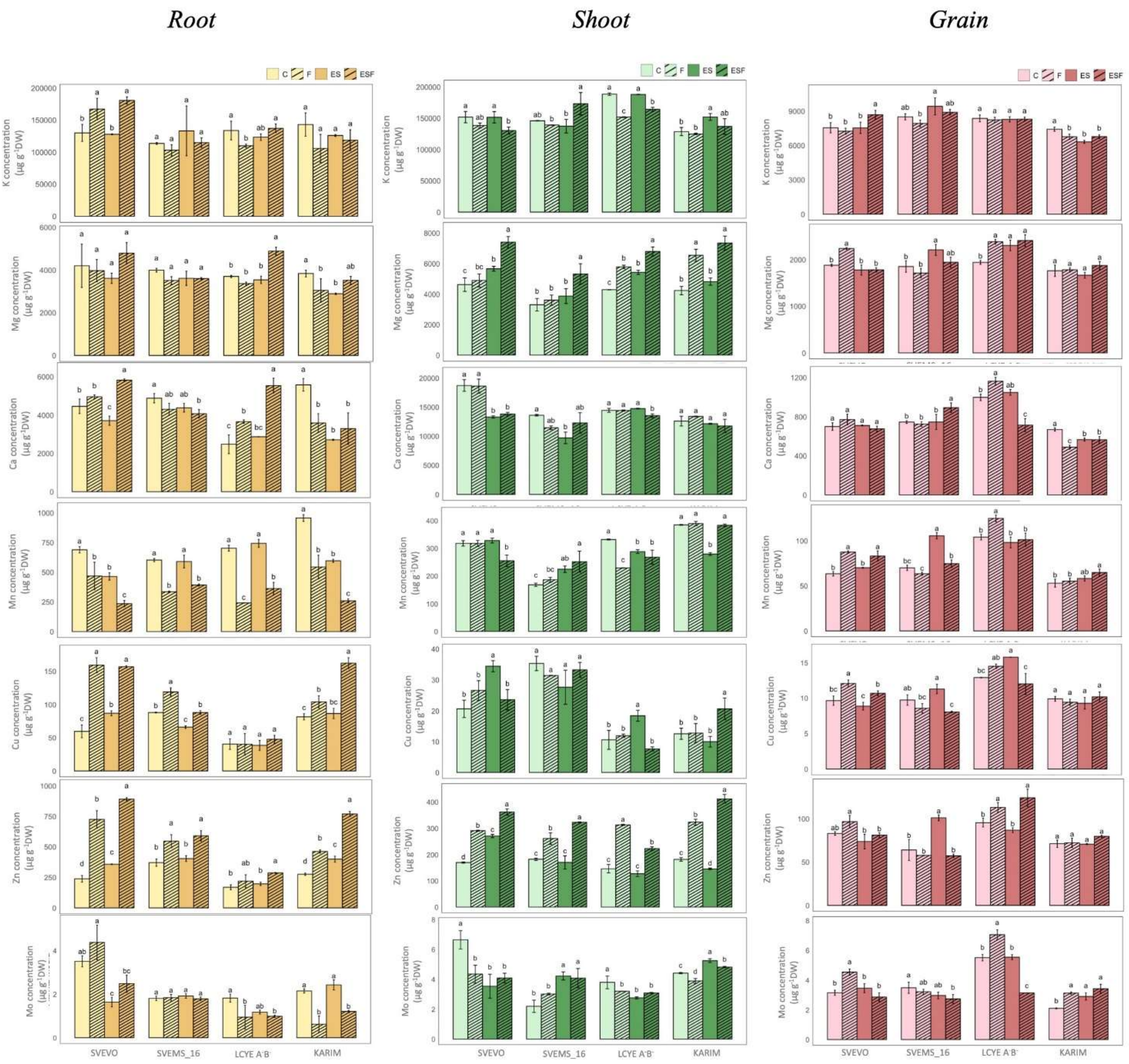

Supplementary Figure 2 (Fig. S2). K, Mg, Ca, Mn, Cu, Zn and Mo concentrations in root and shoot tissues of four durum wheat genotypes grown hydroponically and in grains of the same genotypes grown under greenhouse conditions as described in Fig.1. Statistics as in Figure 2.

Supplementary Table 1. Two-way ANOVA on physiological, morphological and biochemical traits evaluated in 4 genotypes (Svevo, Svems16, LcyE A^-B^-, and Karim) grown in hydroponics.

Two-way ANOVA	Effects	d.f.	SS	MS	F	*p*-value
**Root FW (g plant^{-1})**	Growth condition	3	0.398	0.1326	8.753	$1.63e^{-05}$ ***
	Genotype	3	2.058	0.6859	45.273	< $2e^{-16}$ ***
	Interaction	9	0.100	0.0111	0.753	0.677 **ns**
	Residuals	224	3.393	0.0151		
**Shoot FW (g plant^{-1})**	Growth condition	3	0.156	0.0521	2.764	0.0428 *
	Genotype	3	2.003	0.6677	35.442	< $2e^{-16}$ ***
	Interaction	9	0.110	0.0122	0.650	0.7535 **ns**
	Residuals	224	4.220	0.0188		
**Chlorophyll content (SPAD units)**	Growth condition	3	764	254.55	12.247	1.89 e^{-07} ***
	Genotype	3	221	73.66	3.554	0.0154 *
	Interaction	9	175	19.48	0.937	0.4935 **ns**
	Residuals	224	4656	20.79		
**Root length (cm)**	Growth condition	3	36.85	12.283	41.124	4.35 e^{-11} ***
	Genotype	3	67.99	22.664	75.877	1.23 e^{-14} ***
	Interaction	9	17.89	1.988	6.655	2.60 e^{-05} ***
	Residuals	32	9.56	0.299		
**Surface area (cm^2)**	Growth condition	3	3057	1018.9	53.93	1.30 e^{-12} ***
	Genotype	3	7609	2536.4	134.23	< 2 e^{-16} ***
	Interaction	9	2844	316	16.72	9.44 e^{-10} ***
	Residuals	32	605	18.9		
**Root volume (cm^3)**	Growth condition	3	0.3271	0.10905	37.069	1.59 e^{-10} ***
	Genotype	3	0.5038	0.16795	57.092	6.06 e^{-13} ***
	Interaction	9	0.2408	0.02676	9.097	1.15 e^{-06} ***
	Residuals	32	0.0941	0.00294		
**Average diameter (mm)**	Growth condition	3	0.00718	0.002394	4.175	0.0133 *
	Genotype	3	0.04691	0.015637	27.273	6.03 e^{-09} ***
	Interaction	9	0.00613	0.000681	1.188	0.3361 **ns**
	Residuals	32	0.01835	0.000573		
**root tip number**	Growth condition	3	444692	148231	116.86	< 2 e^{-16} ***
	Genotype	3	1386327	462109	364.31	< 2 e^{-16} ***
	Interaction	9	346790	38532	30.38	3.26 e^{-13} ***
	Residuals	32	40590	1268		
**Root total S concentration (mg g^{-1} DW)**	Growth condition	3	8.563	2.854	47.91	6.15 e^{-12} ***
	Genotype	3	13.805	4.602	77.23	9.62 e^{-15} ***
	Interaction	9	6.192	0.688	11.55	8.25 e^{-08} ***
	Residuals	32	1.907	0.060		
**Shoot total S concentration (mg g^{-1} DW)**	Growth condition	3	13.81	4.603	28.81	3.22 e^{-09} ***
	Genotype	3	81.48	27.159	169.97	< 2 e^{-16} ***
	Interaction	9	15.90	1.766	11.05	1.36 e^{-07} ***
	Residuals	32	5.11	0.160		
**Root thiol concentration (nmol g^{-1} FW)**	Growth condition	3	41167	13772	28.49	3.66 e^{-09} ***
	Genotype	3	26156	8719	18.10	4.76 e^{-07} ***
	Interaction	9	48086	5343	11.09	1.30 e^{-07} ***
	Residuals	32	15413	482		
**Shoot thiol concentration (nmol g^{-1} FW)**	Growth condition	3	10010	3337	2.863	0 0521 **.**
	Genotype	3	10533	3511	3.013	0.0444 *
	Interaction	9	83894	9322	7.999	4.35 e^{-06} ***
	Residuals	32	37291	1165		
**Root ATPS activity (nmol ATP min^{-1} mg prot^{-1})**	Growth condition	3	209.4	69.79	230.4	< 2 e^{-16} ***
	Genotype	3	301.2	100.41	331.5	< 2 e^{-16} ***

	Interaction	9	406.9	45.21	149.2	< 2 e^{-16} ***
	Residuals	32	9.7	0.30		
**Shoot ATPS activity (nmol ATP min^{-1} mg prot^{-1})**	Growth condition	3	289.6	96.53	75.43	1.34 e^{-14} ***
	Genotype	3	90.1	30.02	23.46	3.22 e^{-08} ***
	Interaction	9	603.3	67.03	52.38	< 2 e^{-16} ***
	Residuals	32	41.0	1.28		
**Root OASTL activity (mmol min^{-1} mg prot^{-1})**	Growth condition	3	117	39	44.33	1.68 $^{e-11}$ ***
	Genotype	3	10950	3650	4131.02	< 2 e^{-16} ***
	Interaction	9	5.68	63	71.41	< 2 e^{-16} ***
	Residuals	32	28	1		
**Shoot OASTL activity (mmol min^{-1} mg prot^{-1})**	Growth condition	3	96	32.0	28.71	3.34 e^{-09} ***
	Genotype	3	5601	1867.1	1673.61	< 2 e^{-16} ***
	Interaction	9	248	27.6	24.71	5.62 e^{-12} ***
	Residuals	32	36	1.1		
**PS release rate (µmol g^{-1} FW)**	Growth condition	3	2.1457	0.7152	35.25	2.94 e^{-10} ***
	Genotype	3	1.6094	0.5365	26.44	8.58 e^{-09} ***
	Interaction	9	1.8399	0.2044	10.08	3.81 e^{-07} ***
	Residuals	32	0.6493	0.0203		
**Root Fe concentration (µg g^{-1} DW)**	Growth condition	3	471970	157323	120.65	< 2 e^{-16} ***
	Genotype	3	68999	23000	17.64	6.14 e^{-07} ***
	Interaction	9	385311	42812	32.83	1.09 e^{-13} ***
	Residuals	32	41726	1304		
**Shoot Fe concentration (µg g^{-1} DW)**	Growth condition	3	51336	17112	244.92	< 2 e^{-16} ***
	Genotype	3	95726	31909	456.69	< 2 e^{-16} ***
	Interaction	9	14855	1651	23.62	1.03 e^{-11} ***
	Residuals	32	2236	70		
**Root Cu concentration (µg g^{-1} DW)**	Growth condition	3	20829	6943	118.93	< 2 e^{-16} ***
	Genotype	3	39996	13332	228.37	< 2 e^{-16} ***
	Interaction	9	18681	2076	35.55	3.$^{54e-14}$ ***
	Residuals	32	1868	58		
**Shoot Cu concentration (µg g^{-1} DW)**	Growth condition	3	51	17.1	2.372	0.0887 .
	Genotype	3	3309	1103.0	152.850	< 2 e^{-16} ***
	Interaction	9	741	82.4	11.413	9.42e^{-08} ***
	Residuals	32	231	7.2		
**Root Zn concentration (µg g^{-1} DW)**	Growth condition	3	973396	324465	314.45	< 2 e^{-16} ***
	Genotype	3	773959	257986	250.02	< 2 e^{-16} ***
	Interaction	9	393165	43685	42.34	2.87e^{-15} ***
	Residuals	32	33019	1032		
**Shoot Zn concentration (µg g^{-1} DW)**	Growth condition	3	240696	80232	587.60	< 2 e^{-16} ***
	Genotype	3	38109	12703	93.03	6.89e^{-16} ***
	Interaction	9	66238	7360	53.90	< 2 e^{-16} ***
	Residuals	32	4369	137		
**Root Mo concentration (µg g^{-1} DW)**	Growth condition	3	3.241	1.080	11.48	2.86e^{-05} ***
	Genotype	3	20.980	6.993	74.33	1.64e^{-14} ***
	Interaction	9	17.339	1.927	20.48	6.93e^{-11} ***
	Residuals	32	3.011	0.094		
**Shoot Mo concentration (µg g^{-1} DW)**	Growth condition	3	2.596	0.865	5.869	0.0026 **
	Genotype	3	21.429	7.143	48.451	5.31e^{-12} ***
	Interaction	9	27.317	3.035	20.588	6.45e^{-11} ***
	Residuals	32	4.718	0.147		
**Root Ca concentration (µg g^{-1} DW)**	Growth condition	3	10309991	3436664	29.14	2.83e^{-09} ***
	Genotype	3	9608113	3202704	27.15	6.35e^{-09} ***
	Interaction	9	28409520	3156613	26.76	1.89e^{-12} ***
	Residuals	32	3774501	117953		
**Shoot Ca concentration (µg g^{-1} DW)**	Growth condition	3	49143488	16381163	28.98	3.00e^{-09} ***
	Genotype	3	136650888	45550296	80.59	5.29e^{-15} ***

	Interaction	9	59969880	6663320	11.79	$6.49e^{-08}$ ***
	Residuals	32	18086793	565212		
**Root K concentration ($\mu g\ g^{-1}$ DW)**	Growth condition	3	$1.641e^{+09}$	$5.471e^{+08}$	2.499	0.077263 .
	Genotype	3	$8.505e^{+09}$	$2.835e^{+09}$	12.949	$1.05e^{-05}$ ***
	Interaction	9	$9.664e^{+09}$	$1.074e^{+09}$	4.904	0.000364 ***
	Residuals	32	$7.006e^{+09}$	$2.189e^{+08}$		
**Shoot K concentration ($\mu g\ g^{-1}$ DW)**	Growth condition	3	$2.388e^{+09}$	$7.960e^{+08}$	14.29	$4.42e^{-06}$ ***
	Genotype	3	$9.546e^{+09}$	$3.182e^{+09}$	57.13	$6.01e^{-13}$ ***
	Interaction	9	$5.378e^{+09}$	$5.976e^{+08}$	10.73	$1.90e^{-07}$ ***
	Residuals	32	$1.782e^{+09}$	$5.570e^{+07}$		
**Root Mg concentration ($\mu g\ g^{-1}$ DW)**	Growth condition	3	5100791	1700264	12.390	$1.53e^{-05}$ ***
	Genotype	3	4297473	1432491	10.439	$6.06e^{-05}$ ***
	Interaction	9	3494524	388280	2.829	0.0144 *
	Residuals	32	4391332	137229		
**Shoot Mg concentration ($\mu g\ g^{-1}$ DW)**	Growth condition	3	42651920	14217307	107.081	$< 2\ e^{-16}$ ***
	Genotype	3	24010051	8003350	60.279	$2.92e^{-13}$ ***
	Interaction	9	7511283	834587	6.286	$4.39e^{-05}$ ***
	Residuals	32	4248695	132772		
**Root Mn concentration ($\mu g\ g^{-1}$ DW)**	Growth condition	3	1336279	445426	206.69	$< 2\ e^{-16}$ ***
	Genotype	3	110736	36912	17.13	$8.17e^{-07}$ ***
	Interaction	9	432789	48088	22.31	$2.22e^{-11}$ ***
	Residuals	32	68961	2155		
**Shoot Mn concentration ($\mu g\ g^{-1}$ DW)**	Growth condition	3	3376	1125	5.639	0.00322 **
	Genotype	3	142692	47564	238.301	$< 2\ e^{-16}$ ***
	Interaction	9	61395	6822	34.177	$6.2e^{-14}$ ***
	Residuals	32	6387	200		

Supplementary Table 2. Two-way ANOVA on agronomic traits (thousand-seed weight, number of spikes per plant, spike weight and chlorophyll content 22, 36, 49 and 63 days after transplanting - DAT) and nutrients accumulation (Fe, Cu, Zn, Mo, Ca, K, Mg and Mn) evaluated in 4 genotypes (Svevo, Svems16, LcyE A^-B^-, and Karim) grown in greenhouse under different nutrient availability conditions: C, control; F, Fe-deficiency; ES, supra-optimal S; ESF, supra-optimal S/Fe-deficiency.

**Two-way ANOVA**	**Effects**	**d.f.**	**SS**	**MS**	**F**	***p*-value**
**One thousand seed weight (g seeds^{-1} DW/n° seeds)**	Growth condition	3	5.6	1.85	0.193	0.901 **ns**
	Genotype	3	882.8	294.26	30.687	5.86e^{-14} ***
	Interaction	9	656.3	72.92	7.604	2.52e^{-08} ***
	Residuals	95	911	9.59		
**Spike weight (g^{-1} DW)**	Growth condition	3	1.180	0.3932	2.967	0.0348 *
	Genotype	3	8.549	2.8497	21.506	3.51e^{-11} ***
	Interaction	9	2.214	0.2460	1.856	0.0652 **.**
	Residuals	118	15.636	0.1325		
**Spike number**	Growth condition	3	0.547	0.1822	0.989	0.401 **ns**
	Genotype	3	4.977	1.6592	9.007	2.65 e^{-05} ***
	Interaction	9	0.515	0.0573	0.311	0.970 **ns**
	Residuals	94	17.315	0.1842		
**Chlorophyll content 22 DAT (SPAD unit)**	Growth condition	3	3.40	1.134	4.178	0.0088 **
	Genotype	6	40.84	6.806	25.080	9.36e^{-16} ***
	Interaction	6	22.18	3.697	13.624	3.18e^{-10} ***
	Residuals	71	19.27	0.271		
**Chlorophyll content 36 DAT (SPAD unit)**	Growth condition	3	121.9	40.63	8.6	6.30e^{-05} ***
	Genotype	3	436.9	145.63	30.828	9.32e^{-13} ***
	Interaction	9	321.3	35.70	7.557	1.30e^{-07} ***
	Residuals	69	325.9	4.72		
**Chlorophyll content 49 DAT (SPAD unit)**	Growth condition	3	203.7	67.91	18.29	6.82e^{-09} ***
	Genotype	3	508	169.33	45.59	< 2e^{-16} ***
	Interaction	9	341.6	37.95	10.22	6.40e^{-10} ***
	Residuals	71	263.7	3.71		
**Chlorophyll content 63 DAT (SPAD unit)**	Growth condition	3	14.8	4.93	0.411	0.745473 **ns**
	Genotype	3	456	152.02	12.663	6.41e^{-07} ***
	Interaction	9	429.1	47.68	3.972	0.000294 ***
	Residuals	85	1020.4	12		
**Fe concentration (µg g^{-1} DW)**	Growth condition	3	31	10.3	0.181	0.909 **ns**
	Genotype	3	3706	1235.4	21.599	7.80e^{-08} ***
	Interaction	9	4070	452.3	7.908	4.88e^{-06} ***
	Residuals	32	1830	57.2		
**Cu concentration (µg g^{-1} DW)**	Growth condition	3	9.08	3.03	7.73	0.000508 ***
	Genotype	3	148.30	49.43	126.31	< 2e^{-16} ***
	Interaction	9	53.61	5.96	15.22	3.06e^{-09} ***
	Residuals	32	12.52	0.39		
**Zn concentration (µg g^{-1} DW)**	Growth condition	3	376	125.2	3.932	0.017 *
	Genotype	3	8898	2965.9	93.161	6.76e^{-16} ***
	Interaction	9	7139	793.2	24.914	5.02e^{-12} ***
	Residuals	32	1019	31.8		
**Mo concentration (µg g^{-1} DW)**	Growth condition	3	12.81	4.271	76.97	1.01e^{-14} ***
	Genotype	3	43.75	14.583	262.79	< 2e^{-16} ***
	Interaction	9	19.29	2.144	38.63	1.08e^{-14} ***
	Residuals	32	1.78	0.055		
**Ca concentration**	Growth condition	3	41579	13860	9.03	0.000177 ***

**(µg g^{-1} DW)**	Genotype	3	1038169	346056	225.47	< 2e^{-16} ***
	Interaction	9	404609	44957	29.29	5.42e^{-13} ***
	Residuals	32	49114	1535		
**K concentration (µg g^{-1} DW)**	Growth condition	3	2432804	810935	7.344	0.000703 ***
	Genotype	3	23654975	7884992	71.407	2.87e^{-14} ***
	Interaction	9	6754083	750454	6.796	2.13e^{-05} ***
	Residuals	32	3533566	110424		
**Mg concentration (µg g^{-1} DW)**	Growth condition	3	207582	69194	8.859	0.000202 ***
	Genotype	3	1538913	512971	65.680	9.07e^{-14} ***
	Interaction	9	1105789	122865	15.731	2.03e^{-09} ***
	Residuals	32	249926	7810		
**Mn concentration (µg g^{-1} DW)**	Growth condition	3	870	290	18.92	3.06e^{-07} ***
	Genotype	3	14943	4981	324.75	< 2e^{-16} ***
	Interaction	9	4991	555	36.16	2.78e^{-14} ***
	Residuals	32	491	15		
